# Assessing the sensitivity of urban aquatic nature-based solutions to hydroclimate variability using stable water isotopes

**DOI:** 10.1007/s10661-025-14882-x

**Published:** 2025-12-06

**Authors:** Maria Magdalena Warter, Chris Soulsby, Kati Vierikko, Silvia Martín Muñoz, Daniel Gebler, Mariusz Sojka, Vladimíra Dekan Carreira, Cristina Antunes, Pedro Pinho, Dörthe Tetzlaff

**Affiliations:** 1 Department of Ecohydrology and Biogeochemistry, Leibniz Institute of Freshwater Ecology and Inland Fisheries, Berlin, Germany; 2 Northern Rivers Institute, University of Aberdeen, St. Mary’s Building, Kings College, Old Aberdeen, Scotland; 3 Chair of Water Resources Management and Modeling of Hydrosystems, Technical University Berlin, Berlin, Germany; 4 Finnish Environment Institute, Built Environment Solutions Unit, Helsinki, Finland; 5 Department of Biology, ECOSPHERE Research Group, University of Antwerp, Antwerp, Belgium; 6 Department of Ecology and Environmental Protection, Poznań University of Life Sciences, Poznań, Poland; 7 Department of Land Improvement, Environmental Development and Spatial Management, Poznań University of Life Sciences, Poznań, Poland; 8 cE3c - Center for Ecology, Evolution and Environmental Changes & CHANGE - Global Change and Sustainability Institute, Faculty of Sciences, University of Lisbon, C2, Campo Grande, Lisbon, Portugal; 9 Department of Geography, Humboldt University of Berlin, Berlin, Germany

**Keywords:** Urban water management, Transit times, Urbanization, Climate adaptation, Water ages, Isotope hydrology, Blue infrastructure

## Abstract

Natural and engineered water features, or blue infrastructure are increasingly implemented in cities as a form of water-related nature-based solutions (aquaNBS), to address ecological and hydrological challenges that threaten urban biodiversity and water security. Nevertheless, the combination of impacts from climate change, multi-faceted consequences of past management, current urban expansion, population growth, and overall urban ecosystem complexity makes it challenging to evaluate the hydrological function of these aquaNBS, and their sensitivity to hydroclimatic and other environmental changes. To enhance adaptation capacity of aquaNBS towards multiple urban and climatic stressors, it is crucial to understand the main hydrologic processes, as well as hydroclimate influences, that determine the functioning of aquaNBS. Stable water isotopes have proven to be a valuable tool in providing integrated understanding of hydrologic functioning over extended spatial scales. While higher frequency isotope data is usually most informative, even limited isotopic data can aid hydrological characterization. We conducted seasonal sampling over the period of one year in 2023/2024, across a major hydroclimate gradient across four European cities (Poznań, Berlin, Antwerp, Lisbon). The goal was to identify the dominant physical processes (in terms of water sources, dominant flow paths, and age proxies) linked to the main hydroclimate factors along a continental climate gradient. Comparative analyses of local stable water isotope signatures from different aquaNBS types (i.e., streams, ponds) revealed the strong influence of local hydroclimate, as well as varying water source contributions and mixing processes. The application of transit time proxies, such as tracer damping and young water fraction estimations, suggests ponds to be more sensitive to hydroclimate changes, as evidenced by the strong seasonality in evaporative enrichment and high fractions of young water contributions. In contrast, most streams indicated greater mixing of water sources and longer transit times, suggesting greater resilience to hydroclimate variability. In addition, a comparison between seasonally sampled data and monthly sampling for selected locations in Berlin showed that even relatively coarse temporal data collection, but with more extensive spatial coverage, can be sufficient and still insightful for broader hydrologic characterizations of aquaNBS at larger scales.

**Supplementary Information:**

The online version contains supplementary material available at 10.1007/s10661-025-14882-x.

## Introduction

The multifaceted impacts of climate change and urban development increasingly challenge urban water management. Today, cities have evolved into highly engineered environments, where the natural water cycle has been profoundly altered (Gessner et al., 2014; Li et al., 2020; Walsh et al., 2005). Extensive impervious surfaces on roads and buildings limit infiltration and accelerate runoff, while widespread subsurface storm drain networks can disconnect surface water from groundwater systems, conveying runoff directly into streams, lakes or ponds with little or no attenuation or treatment (Bonneau et al., 2018; Burns et al., 2012; Ress & James, 2020). These hydrologic alterations have generally led to “flashier” urban flow regimes, with larger storm runoff volumes and higher, more frequent peak flow responses to extreme precipitation events. At the same time, the capacity of urban landscapes to store, filter, and slowly release water is substantially reduced, thereby increasing flood risk and reducing recharge (Golden & Hoghooghi, 2018; Oswald et al., 2023; Walsh et al., 2012; Yang et al., 2011). Temperature extremes and increased evapotranspiration from urban heat island effects further contribute to water stress, especially during dry periods, leading to more intermittent rivers and ephemeral streams, further reduced groundwater recharge and vegetation water stress (Kuhlemann et al., 2020; Ring et al., 2024; Warter et al., 2024a). Given the complexity of the urban water cycle and its different components of engineered and natural hydrology, urban freshwater systems are experiencing chronic ecological, chemical and hydrological stress (Marx et al., 2023; Numberger et al., 2022; Richardson & Soloviev, 2021). These challenges to sustainable urban water management call for more adaptive approaches beside traditional grey infrastructure of piped storm drains, to ameliorate the negative impacts of urbanization and climate change on urban freshwater resources and aiding the transition towards more resilient urban environments (Bush & Doyon, 2019; Davis & Naumann, 2017; Krueger et al., 2020; Wild et al., 2024).

Blue infrastructure, such as urban wetlands, ponds, or restored streams and floodplains, are increasingly utilized as water-related (or aquatic) nature-based solutions (aquaNBS), substituting traditional grey infrastructure. By harnessing the multifunctionality of blue infrastructure in urban landscapes, a wide range of environmental and societal challenges related to climate change, human health and well-being, biodiversity and water security can be addressed (Chowdhury et al., 2025; Kabisch et al., 2016; Pinho et al., 2023; van Rees et al., 2023). The recognition of the value of blue infrastructure as aquaNBS for enhancing urban ecosystem services and supporting sustainable urban water management has led to widespread ecological restoration efforts of degraded urban freshwater ecosystems (Everard & Moggridge, 2012; Hack & Schröter, 2020; Lammers et al., 2020). By restoring and re-establishing near-natural hydrological functioning, aquaNBS are expected to slow runoff, enhance infiltration and storage, reconnect surface and subsurface flows, and help to reduce pollutant loads (Hack & Schröter, 2020; Williams & Filoso, 2023).

The effectiveness of aquaNBS is governed by local hydrological processes (e.g., water source partitioning, surface and subsurface flow paths, groundwater-surface water interactions, and water residence times) and their linkage with hydroclimate and the urban matrix. As aquaNBS can comprise a broad range of catchment interventions, their design and implementation require not only an understanding of these underlying hydrological processes and boundary conditions, but also of their potential evolution under variable and changing climatic conditions. As such, the lack of empirical evidence of the role of hydrological processes in the function and effectiveness of different types of aquaNBS is critical for their successful long-term implementation and management (Lalonde et al., 2024; Pinho et al., 2023). This poses the risk that aquaNBS may not produce the intended benefits or lead to unintended ecosystem responses with undesirable consequences for water quality, human health and biodiversity (Lalonde et al., 2024). Considering the speed of global change, the resilience and ability of aquaNBS to evolve and withstand changing climate conditions not only depend on structural design, but also on their ability to cope with short-term climatic perturbations (e.g., heavy rainfall, droughts) as well as long-term shifts in hydroclimate regimes. Therefore, there is a clear need to better understand how urban water sources and hydroclimate conditions systems affect aquaNBS functioning.

Characterizing the dominant hydrological processes in built-up areas, and of aquaNBS specifically, remains one of the key challenges of urban hydrological research (Oswald et al., 2023). In particular, inter-comparisons of cities across different climate and geographic gradients are important, but difficult, as high-frequency sampling over extended spatial scales is often logistically demanding and impractical. As naturally occurring tracers of the water cycle, the stable water isotope ratios of oxygen (δ^18^O) and hydrogen (δ^2^H) can be a useful integrating tool to differentiate contrasting water sources across catchment, regional and global scales and to characterize fundamental hydrological processes and water fluxes (Ehleringer et al., 2016; Jefferson et al., 2015; Tetzlaff et al., 2015). Isotopic signatures of different water sources (i.e., precipitation, groundwater, runoff) allow to investigate connectivity between landscapes and freshwater ecosystems, as well as climate-water-ecosystem interactions between natural and anthropogenic systems (Kendall & McDonnell, 1998; Kirchner, 2016; Soulsby et al., 2015).

Regional assessments of urban streamflow sources (Kuhlemann et al., 2021; Marx et al., 2021), groundwater contributions (Vystavna et al., 2019), urban water supply dynamics (Bhuiyan et al., 2023; Jameel et al., 2018), and the impacts of urbanization and climate stress in anthropogenically impacted catchments (Kuhlemann et al., 2020; Soulsby et al., 2014; Warter et al., 2024a) have highlighted the value of spatially distributed sampling in cities. In addition, by using relatively simple transit time proxies, such as young water fractions (e.g., the proportion of a water body that is less than ~ 3 months old), mean transit times can be assessed and used to contextualize local hydroclimate, landscape controls and water sources (Hrachowitz et al., 2010; Kirchner, 2016; Soulsby et al., 2015; Von Freyberg et al., 2018a, 2018b). Especially in urban watersheds, understanding water transit times is important for assessing dominant streamflow generation processes and evaluate potential sensitivities to hydroclimate changes (Morales & Oswald, 2020; Warter et al., 2024b).

Clearly, leveraging existing urban blue infrastructures for aquaNBS requires an understanding of local water source dynamics and key hydroclimate drivers. Therefore, we used stable water isotopes as an integrated lens through which to understand the role of hydrology in urban aquaNBS and to provide an urgently needed evidence basis of the key hydroclimate and hydrological processes that influence the hydrological functioning of aquaNBS. With a geographical focus along a strong hydroclimatic gradient of four European Cities — Poznań (Poland), Berlin (Germany), Antwerp (Belgium), and Lisbon (Portugal), we undertook low-frequency sampling of stable water isotopes in different types of aquaNBS (streams and ponds) at — in total — 48 locations between February 2023 and March 2024. Concurrently, we used transit time proxies such as young water fractions to assess flow pathways and transit times across urban aquaNBS. This also allowed us to evaluate sampling efficiency and suitability of such low-frequency sampling for broader hydrological characterization. More specifically, we investigated the following objectives: (i) Identify the main water sources and flow paths in urban aquaNBS across a hydroclimate gradient in Europe; (ii) Use low-frequency water stable isotope data to infer differences in water residence times across contrasting European cities through simple travel time proxies such as young water fractions; and (iii) Discuss the value of tracer-based diagnostics to assess hydrological processes and the influence of future hydroclimate variability in different aquaNBS.

## Methodology

### Study sites

**Fig. 1:**
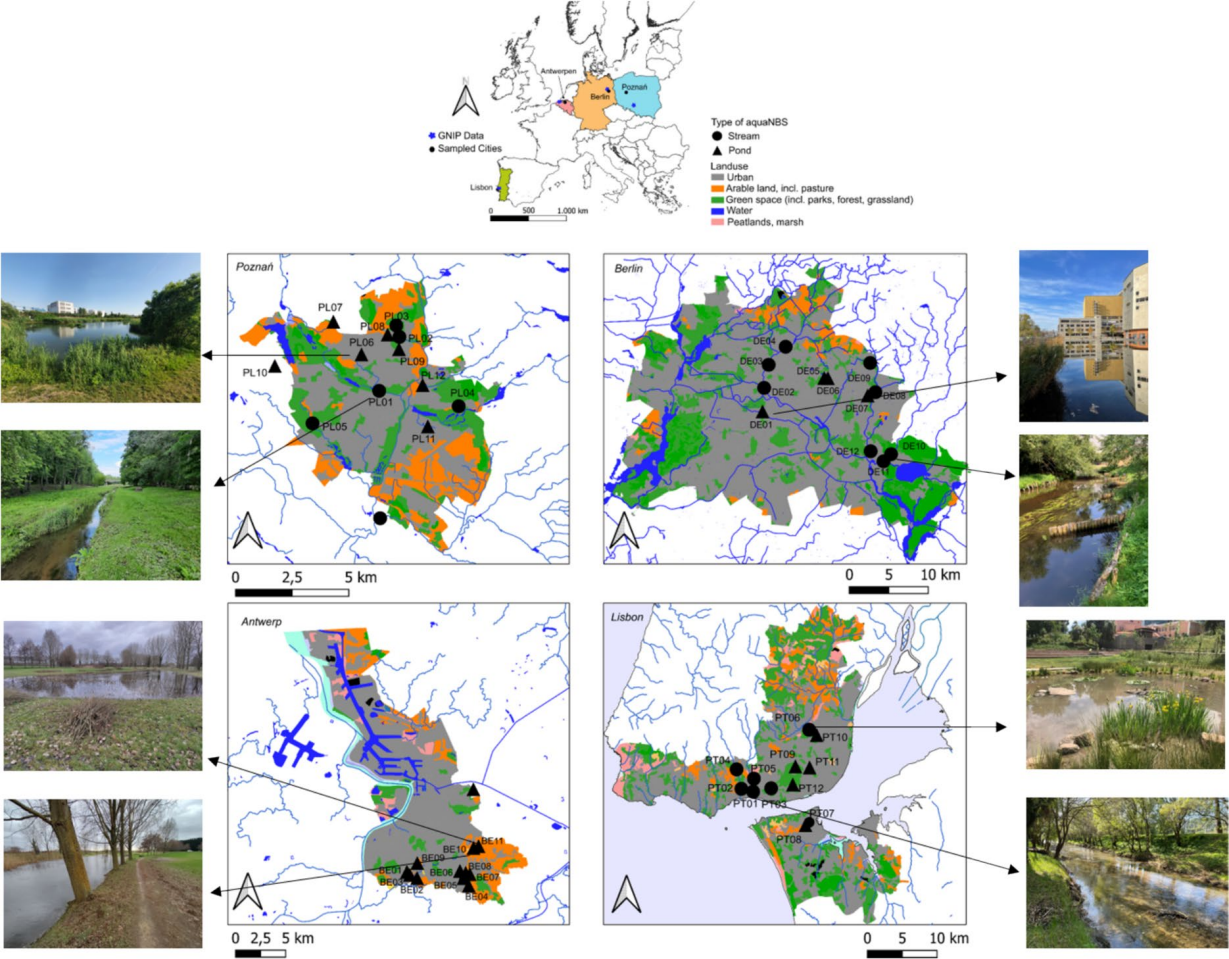
Overview of urban case study sites in each city showing land use distribution (incl. urban, green space, arable land, and water bodies). Sample points are denoted with symbols (triangle = pond, filled circle = stream). Pictures of two representative sites in each city are shown

Four urban case study cities were selected – Poznań, Berlin, Antwerp and Lisbon – set in different hydroclimate contexts across Europe: continental (Berlin, Poznań), oceanic (Antwerp) and Mediterranean (Lisbon). Site selection in each city was done using a stratified random sampling approach (12 locations per city), with the goal to have a wide geographic representation of different types of aquaNBS within each urbanized setting. We aimed for diverse urban blue infrastructural features that include artificial and natural pond and stream-based aquaNBS (Fig. 1), with different sizes (pond water volume), permanence (perennial, intermittent) and stream orders (2nd, 3rd order). Hydroclimate and land use characteristics of the study cities are summarized in Table 1 (see Table S2 in the Supplementary Material for more information on individual sample sites or Szoszkiewicz et al., 2024).

**Table 1:** Site characteristics of the study locations including mean annual precipitation (in mm/year, long-term average), mean elevation (in meters above sea level (m.a.s.l.), mean annual temperature (T, in °C), mean annual potential evapotranspiration (PET, in mm/yr), the number of samples sites in each city as well as the dominant land use (in %) in each city (Source: Copernicus CORINE Land Cover 2018, Europe, 6-yearly)

City	Annual precipitation (mm/yr)	Elevation (masl)	Mean T (°C)	Mean PET (mm/yr)	Nr. sampled sites	Dominant land use (%)		
						*Arable*^*°*^	Green^*^	*Urban*^+^
Poznań	539	85	9.4	450–500	Stream: 6 Pond: 6	25	21	45
Berlin	577	47	12.0	700	Stream: 8 Pond: 4	3	30	55
Antwerp	830	9	11.0	500–600	Streams: na Ponds: 12	8	11	60
Lisbon	774	51	21.0	1100	Stream: 7 Pond: 5	5	26	42

*Green includes urban green spaces, grassland and forests (mixed, broadleaf, coniferous)

+Urban includes all urban fabric, roads, industrial units, airports and port areas

°Arable includes all irrigated and non-irrigated agricultural land and pastures

Briefly, the city of Poznań (N 52°24′45″, E 16°55′45″) is located in the lowland central-western part of Poland, in the Warta River basin. The city is rich in small and large urban blue and green spaces and forests. Poznań’s climate is considered a temperate oceanic climate, characterized by cold winters and warmer summers, with mean annual precipitation of around 539 mm/yr (1991–2020 average) and a mean annual PET between 450 and 500 mm/yr (1991–2020 average) (Bochenek et al., 2024). In Poznań, a mixture of small 2nd or 3rd order perennial and intermittent streams and ponds were sampled, which primarily provide a means of urban flash flood regulation. Ponds in the city ranged in size from 750 m^2^ to 16,000 m^2^, mainly serving as rain water retention ponds from urban runoff.

The city of Berlin (N 52°31′16″, E 13°24′17″) is located in the NE of Germany. The climate is continental, bordering a humid oceanic climate. The central area of Berlin is highly urbanized, with large areas of contiguous urban green space and forest (up to 30%). The city has an extensive network of blue infrastructure with first and second order streams and a large number of small surface water bodies (around 700), ranging from small ponds to larger lakes. Annual precipitation is approximately 577 mm/yr (1981–2020 average) (German Weather Service, DWD, 2023), distributed throughout the year as frequent, low-intensity frontal winter rains and infrequent heavy convective summer storms. Evapotranspiration often exceeds annual precipitation inputs (PET ⁓700 mm/yr). A mixture of perennial and intermittent 2nd-order streams and artificial medium size ponds (12,000–40,000 m^2^) were sampled. Each pond performs slightly different functions — from pure rainwater retention to semi-natural swimming ponds and wetland habitats, thus representing a variety of blue urban infrastructure.

Due to its proximity to the Atlantic, the city of Antwerp (N 51°13′32″, E 4°14′50″) has a strong oceanic climate, characterized by cool winters and warm summers, and frequent, though light, precipitation throughout the year. Annual precipitation is around 830 mm/yr, while the mean annual PET is 500–600 mm/yr. The city has a dense network of artificial channels and ponds to support water retention and urban storm runoff capture. As a result, in Antwerp, only rainwater retention ponds were sampled, ranging in size from 153 m^2^ to 4.281 m^2^. Small to medium rainwater retention systems are extensively used throughout the city and embedded in the urban matrix due to water regulation laws requiring new developments to retain and infiltrate runoff water on-site.

Finally, Lisbon (N 38°42′25″, W 9°08′21″) represents a typical Mediterranean climate, where most rainfall occurs in winter and spring, whereas summers are usually hot and dry. Mean annual precipitation is around 770 mm/yr over an average of 78 rain days. Similar to Berlin, potential evapotranspiration vastly exceeds annual precipitation, with an average PET of > 1000 mm/yr. The city of Lisbon is highly urbanized, though extended urban green spaces and forested areas (up to 40%) characterize the city. We sampled a mixture of 3rd-order perennial and intermittent streams and small ponds throughout the cities of Lisbon and Almada. Most of the streams were within forested areas, primarily supporting flash flood regulation. Ponds varied in size (280 m^2^ to 8000 m^2^) and function, serving as biodiversity hotspots and recreational sites as well as supporting flash flood management. It should be noted that Berlin, Antwerp, and Poznań are closer in latitude and distance than Lisbon, which is much farther south, thus climatic differences will be more pronounced.

### Sampling strategy

Seasonal grab sampling was undertaken in each city between February 2023 and March 2024. Although samples were not always collected in the same month, they roughly correspond to a seasonal schedule (see Table S1 in the Supplementary Material). This was considered the most efficient sampling strategy as part of such a larger geographical assessment over a short timescale. In Berlin, additional monthly sampling was conducted simultaneously at all locations. Samples were subsequently all sent to Berlin, Germany, for laboratory analysis to ensure quality control and limit errors resulting from different measuring techniques and equipment uncertainties. All liquid samples were filtered (0.2-µm cellulose acetate) and decanted into 1.5 mL vials. Samples were analyzed for δ^18^O and δ^2^H using a Picarro L2130-i cavity ring-down water isotope analyzer (Picarro Inc., Santa Clara, CA, USA). The Vienna Standard Mean Ocean Water (VSMOW) was used as a reference. Analytical precision was 0.05 ‰ standard deviation for δ^18^O and 0.18‰ for δ^2^H.

### Data

#### Hydroclimate

Climate data used in this study — daily precipitation, air temperature and relative humidity — were downloaded for the sample period from open access sources in each country. In Germany, data were obtained from the German Weather Service (DWD) for a weather station in Berlin Dahlem (DWD, 2024). In Belgium, daily precipitation and air temperature were obtained from the Flemish Weather service at a station in Wilrijk, approximately 10 km south of Antwerp (Vlaanderen, 2024). Relative humidity was obtained from a station in Herentals, approximately 30 km west of Antwerp. In Poland, data were obtained from the Institute of Meteorology and Water Management for a station in Poznań-Lawica (IMGW-PIB, 2024). In Lisbon, data were obtained from SNIRH (Sistema Nacional de Informação de Recursos Hidricos) for a station in Monte Caparica, Almada, approximately 12 km south of Lisbon (SNIRH, 2024).

#### Stable water isotopes

Global precipitation isotopes (δ^18^O and δ^2^H) for each city were obtained through the Global Network of Isotopes in Precipitation (GNIP), provided by the International Atomic Energy Agency (IAEA) (IAEA & MO, 2024) (see Locations in Fig. 1). GNIP provides mean monthly values of meteoric precipitation, precipitation amounts and mean monthly temperature measurements. In each country, we selected a GNIP station closest to the study city. In Poland, a GNIP station in Wroclaw, approximately 180 km south of Poznań, had monthly data available for precipitation and snow from 2004 to 2009. In Berlin, a station within the city had data from 1978 to 2021 for rainfall and snow. In Belgium, only one station had data available, but corresponding precipitation amounts were not recorded. Instead, a station in the Netherlands (Gilze Rijn) was chosen, approximately 70 km northeast of Antwerp, where data were available from 1981 to 1988. In Portugal, a station approximately 9 km from the Lisbon had data available from 2003 to 2017. It should be noted that while precipitation isotope data was not contemporaneous to the sampling period, seasonal variabilities were broadly represented. In all cities, multi-year data was available on a monthly basis. For this study, it was deemed important to have multi-year isotope data from local stations rather than estimations of annual average means, such as given by the Online Isotopes in Precipitation Calculator (Bowen, 2025).

Globally, the stable isotopic composition of terrestrial waters is characterized by the Global Meteoric Water line (GMWL) (Craig et al., 1963). On a local level, meteoric waters often show a more variable composition, resulting in Local Meteoric Water Lines (LMWL). Using δ^18^O-δ^2^H relationship of meteoric precipitation in each city, LMWLs were defined for each city as follows:

1 $$\begin{array}{cc}LMWL _{Poznan}: {\delta }^{2}H= {7.36*\delta }^{18}O+7.65& (\mathrm{R}^2=0.95)\end{array}$$

2 $$\begin{array}{cc}LMWL _{Berlin}: {\delta }^{2}H=7.80*{\delta }^{18}O+7.11& (\mathrm{R}^2=0.94)\end{array}$$

3 $$\begin{array}{cc}LMWL _{Antwerp}: {\delta }^{2}H=6.86*{\delta }^{18}O+2.14 & (\mathrm{R}^2=0.94)\end{array}$$

4 $$\begin{array}{cc}LMWL _{Lisbon}: {\delta }^{2}H=6.94{*\delta }^{18}O+7.77 & (\mathrm{R}^2=0.96)\end{array}$$

To assess evaporation effects on surface water isotopic composition, we also calculated the line-conditioned excess (lc-excess), which indicates the deviation of the relationship between δ^2^H and δ^18^O from that of precipitation and the relevant meteoric water line (Landwehr & Coplen, 2006) as well as deuterium excess (d-excess = δ^2^H – 8* δ^18^O), which reflects the degree of evaporation of water sources. A lower d-excess indicates greater evaporation degree (Dansgaard, 1964), while a lower lc-excess also indicates a water source affected by evaporative fractionation (Landwehr & Coplen, 2006).

Local Evaporation Lines (LEL) were constructed using linear least squares regression of surface water isotope samples from each city. Water bodies undergoing evaporation will exhibit distinctive trends in their isotopic ratios away from the precipitation input and the LMWL, with values typically exhibiting strong linear correlation. They form the characteristic LEL, which deviates from and typically lies below the LMWL. By extrapolating the LEL to the intersection with the LMWL, the surface water origin (input water) can be estimated, and source water pathways — from the origin of atmospheric moisture to evapotranspiration, runoff mechanisms and groundwater recharge — can be assessed (Benettin et al., 2018; Evaristo et al., 2015; Zanazzi et al., 2020).

#### Transit time proxies

Precipitation data from GNIP and sampled surface water isotope data were used to estimate two transit time proxies (TTP) — the damping ratio (DR) and the young water fraction (F_yw_). The damping ratio is derived from the ratio of the coefficient of variation of δ^18^O or δ^2^H in streamflow samples to that of δ^18^O or δ^2^H in precipitation. It serves as a semi-quantitative proxy measure to assess general trends and distributions of transit times, with lower damping ratios and higher young water fractions indicating greater influence of more recent precipitation. These can be useful for semi-quantitative characterization of transit times across multiple catchments and larger geographic gradients (Tetzlaff et al., 2009). The DR is described as:

5 $$DR=\frac{CV of {\delta }^{18}O\ or\ {\delta }^{2}H\ in\ streamflow}{CV\ of\ {\delta }^{18}O\ or\ {\delta }^{2}H\ in\ precipiation}$$

where CV represents the coefficient of variation of either δ^18^O or δ^2^H in stream water and precipitation. In general, a higher DR equates to less damping and thus lower transit times, whereas smaller ratios indicate greater damping of precipitation isotopes and thus longer transit times (Tetzlaff et al., 2009). Small ratios have also been shown to be related to higher internal catchment storage volumes involved in greater mixing and have an inverse relationship with mean transit times (Soulsby & Tetzlaff, 2008).

In addition, estimations of young water fractions (F_yw_) can provide indications of water ages and transit time behavior. Young water fractions are considered the fraction of water less than 2–3 months old (Kirchner, 2016). The method developed by Kirchner (2016) uses simple sine-wave fitting and iteratively reweighted least squares regression (IRLS) of tracer input and output. We used volume-weighted observed precipitation isotopes and sampled surface water isotopes. Through comparison of sine-wave fitted amplitudes of precipitation and stream/pond isotopes, F_yw_ can be directly estimated from the amplitudes of seasonal cycles. One of the major benefits of this method is that data requirements are modest and it can be performed on sparse and irregularly sampled data sets, making it an ideal candidate for large multi-site datasets of coarser temporal resolution (Kirchner, 2016). Young water fractions were computed as outlined in Kirchner (2016) using public code by Von Freyberg et al., (2018a, 2018b). All analyses were done using R Studio v.3.4. Generally, higher frequency data (i.e., monthly or daily) significantly reduces uncertainty in DR and F_yw_ estimations. However, in case of sampling constraints, seasonal isotope values can give an initial characterization of broader patterns related to water transit behavior.

## Results

### Evaporation and seasonality of aquaNBS across European cities

**Fig. 2:**
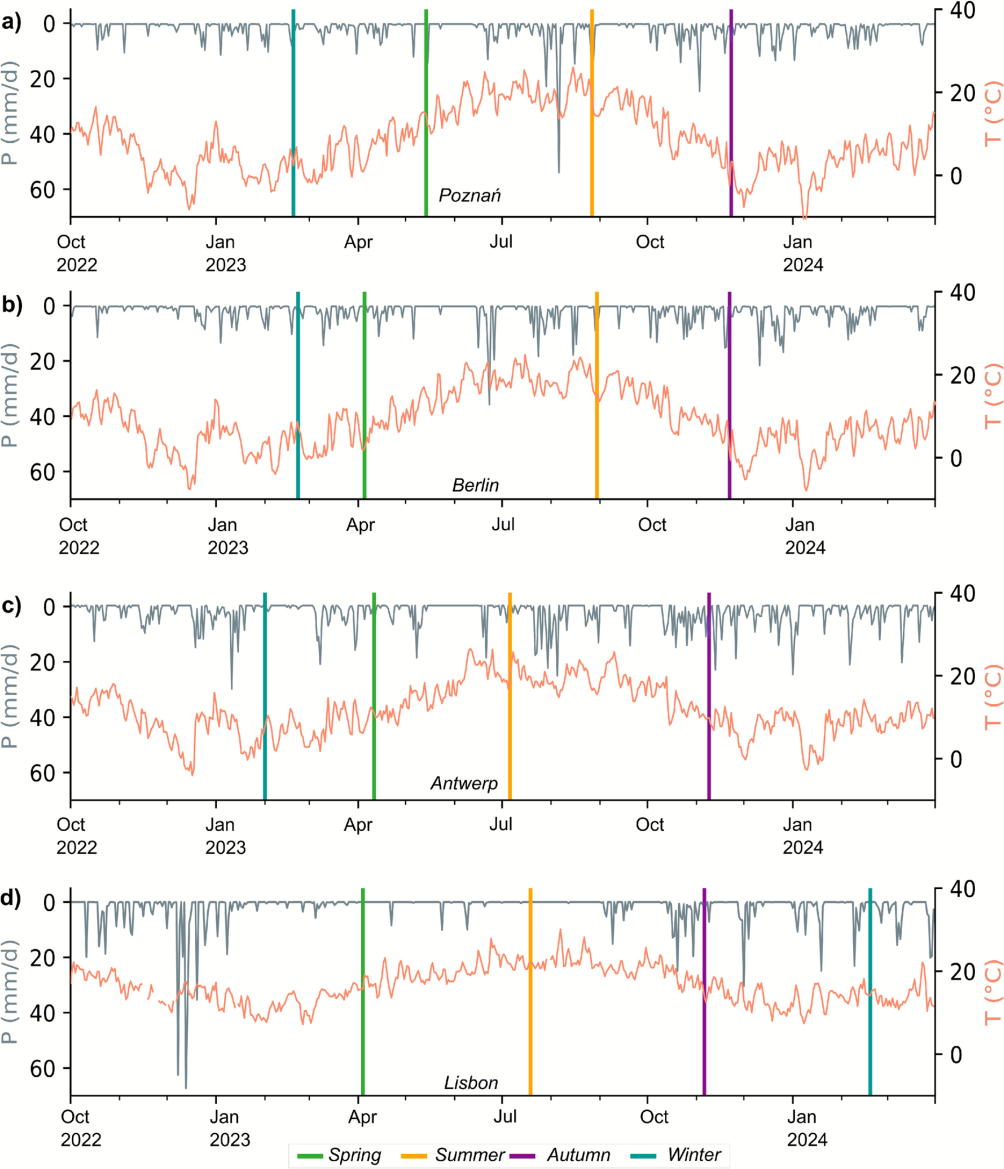
Daily precipitation (in mm, grey) and mean daily temperature (in °C, red) for the period between October 2022 to March 2024 for (**a**) Poznań, (**b**) Berlin, (**c**) Antwerp, and (**d**) Lisbon. Sample dates are indicated with colored horizontal bars

In Poznań, total annual precipitation for 2023 was 710 mm/yr, of which ⁓30% fell between June and August (⁓260 mm). Annual mean temperature was 10.8 °C (Fig. 2a). In Berlin, late 2022 was relatively dry, with < 80 mm between October and December. However, 2023 was an overall wet year, with total annual rainfall of 776.8 mm/yr, which was ⁓30% above of the long-term average (Fig. 2b). Characteristic of the oceanic climate, Antwerp received a total of 980 mm of precipitation, with frequent and intense rainfall in summer and autumn (> 100 mm/month) (Fig. 2c). In Lisbon, late 2022 was relatively wet, with more than 300 mm of rain between October 2022 and January 2023. This was then followed by a dry spring and summer with < 30 mm/month between March and October (Fig. 2d).

**Fig. 3:**
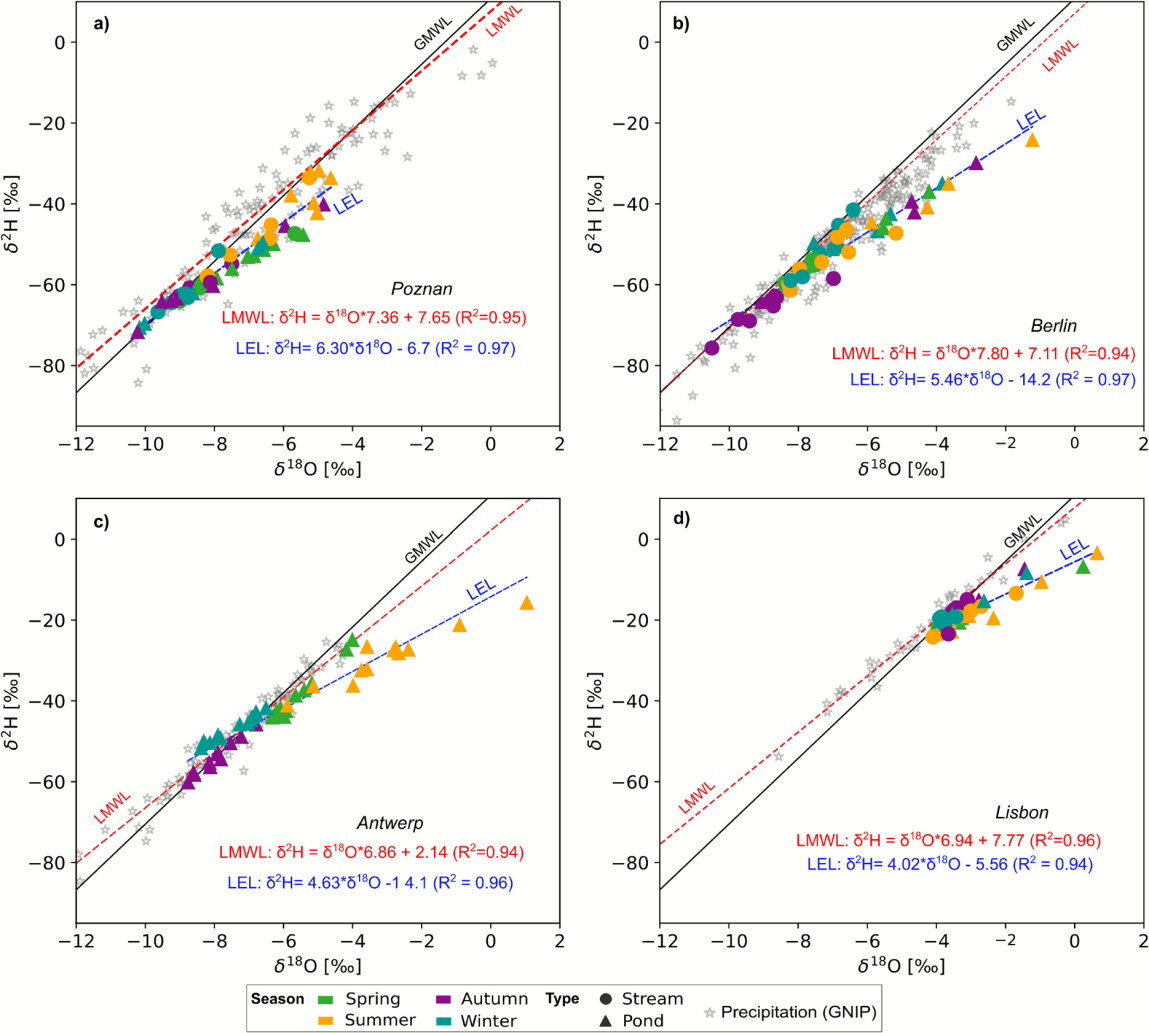
Dual isotope plots of sampled stable water isotopes for different seasons from different aquaNBS (streams or ponds) in (**a**) Poznań, (**b**) Berlin, (**c**) Antwerp, and (**d**) Lisbon. Global (black line) (GMWL) and local (red dashed line) meteoric water lines are indicated. Symbols denote the different type of aquaNBS (stream = filled circle; pond = triangle) and colors denote seasons*. *Local evaporation lines are shown in blue for each city*.* Meteoric precipitation is indicated in grey (from GNIP).

Table 2 reports a descriptive summary of mean δ-values of historic meteoric precipitation and contemporary surface water samples in each city. Across all cities, isotope signatures showed distinct seasonal patterns, with more depleted signatures during winter and progressive enrichment during summer, indicating that pond-based aquaNBS were more susceptible to higher evaporation across all cities. Least squares regression of local GNIP precipitation data resulted in highly significant local meteoric water lines (LMWL) in each city, characteristic of the local hydroclimate (Fig. 3). The similar slopes of the LMWL in Berlin (7.8) and Poznań (7.36) compared to the GMWL (8.0), indicate the absence of complex kinetic fractionation processes affecting meteoric precipitation. Conversely, the lower slopes in Antwerp (6.8) and Lisbon (6.9) highlight the regional differences in moisture cycling in coastal areas and a difference in water vapor sources and seasonal modes of precipitation, explaining the lower slopes.

**Table 2:** Summary descriptive statistics of mean historic δ^18^O and δ^2^H concentrations and d-excess for meteoric precipitation from GNIP, and current samples from aquaNBS sites (stream and pond) in each city taken between 2023 and 2024.

City	Precipitation*			*Streams*			*Ponds*			
	δ^18^O (‰)	δ^2^H (‰)	d-excess (‰)	δ^18^O (‰)	δ^2^H (‰)	d-excess (‰)	δ^18^O (‰)		δ^2^H (‰)	d-excess (‰)
Poznań	− 6.6	− 42.8	11.3 (± 7.6)	− 8.1	− 57.5	7.23 (± 2.8)	− 7.2	− 52.4		5.3 (± 4.3)
Berlin	− 6.9	− 50.3	5.5 (± 3.8)	− 7.7	− 56.6	5.5 (± 3.1)	− 5.1	− 41.9		− 1.5 (± 5.9)
Antwerp	− 7.3	− 47.9	10.4 (± 3.5)	n/a	n/a	-	− 5.9	− 41.7		5.8 (± 7.9)
Lisbon	− 4.3	− 22.1	12.5 (± 2.8)	− 2.4	− 19.5	8.4 (± 2.2)	− 3.5	− 15.2		3.8 (± 5.9)

*Sampling period for meteoric precipitation from GNIPs: Poznań: 2004–2009; Berlin: 1978–2021; Antwerp: 1981–1987; Lisbon: 2003–2017

In Poznań, the isotopic composition of surface waters showed moderate seasonal variability, with δ^18^O values ranging from − 10.2 ‰ to − 4.6‰ and δ^2^H values from − 71.6‰ to − 31.6‰ (Fig. 3a). The more isotopically depleted signatures in stream sites in autumn and winter indicate an influence of depleted winter precipitation on streamflow. Even in the summer, only moderate evaporative enrichment was observed, as inferred from most stream samples plotting close to the GMLW. D-excess values ranged from − 4.1‰ to + 11.4‰, with most negative values in spring. Overall, the sample placement relative to local and global meteoric water lines was characteristic of the mid-latitude continental climate of the Poznań region, with highly seasonal precipitation inputs and moderate evaporation influence during warmer seasons. The LEL intersection with the GMWL further indicates that surface water contributions likely originated from cold-season precipitation and/or cold-season recharge.

The largest variability across all water samples was observed in Berlin (Fig. 3b). A clear distinction was evident between stream and pond samples, with pond samples skewed towards more enriched δ^18^O and δ^2^H signatures, and strongest enrichment during the summer. In Antwerp, pond samples exhibited similar patterns, with more enriched and fractionated signals in summer (Fig. 3c). D-excess values ranged from − 14.3‰ to + 9.7‰ and − 23.9 ‰ to + 16.5‰ in Berlin and Antwerp, respectively, with the most negative d-excess values observed in ponds in summer in both cities, confirming significant evaporative fractionation. Stream samples in Berlin largely plot along the LMWL, with more depleted values observed in autumn and winter. The LEL intersections with the LMWLs indicate that cool-season precipitation and recharge were key water sources driving streamflow and groundwater recharge, especially in winter. In Antwerp, spring, autumn, and winter, samples exhibited overall more depleted signatures, plotting largely along the LMWL, suggesting that the evaporated signal in summer is flushed from the ponds in other seasons, which would indicate a flow-through groundwater source in these ponds.

In contrast, Lisbon showed a more limited variability and overall more enriched signatures, compared to the continental climates of Berlin and Poznań further north. This corresponds to the Mediterranean climate with its strong Atlantic influence (Fig. 3d). More enriched signatures were observed primarily in summer samples of ponds as well as a few stream samples, which were collected in late 2023 and early 2024 following a relatively dry winter. D-excess values ranged from − 8.7‰ to + 10.5‰. The higher intersection of the LEL (− 3.8‰) with the LMWL highlights the overall influence of the naturally more enriched precipitation from oceanic water vapor and shallow water storage (i.e., soil water, shallow groundwater, wetlands). A local groundwater sample also showed a more enriched signature (δ^18^O =  − 4.1‰ and δ^2^H =  − 22.9‰), similar to the stream samples taken in autumn, winter and spring, suggesting a seasonal connection to local groundwater.

### Transit time proxies across urban aquaNBS

**Fig. 4:**
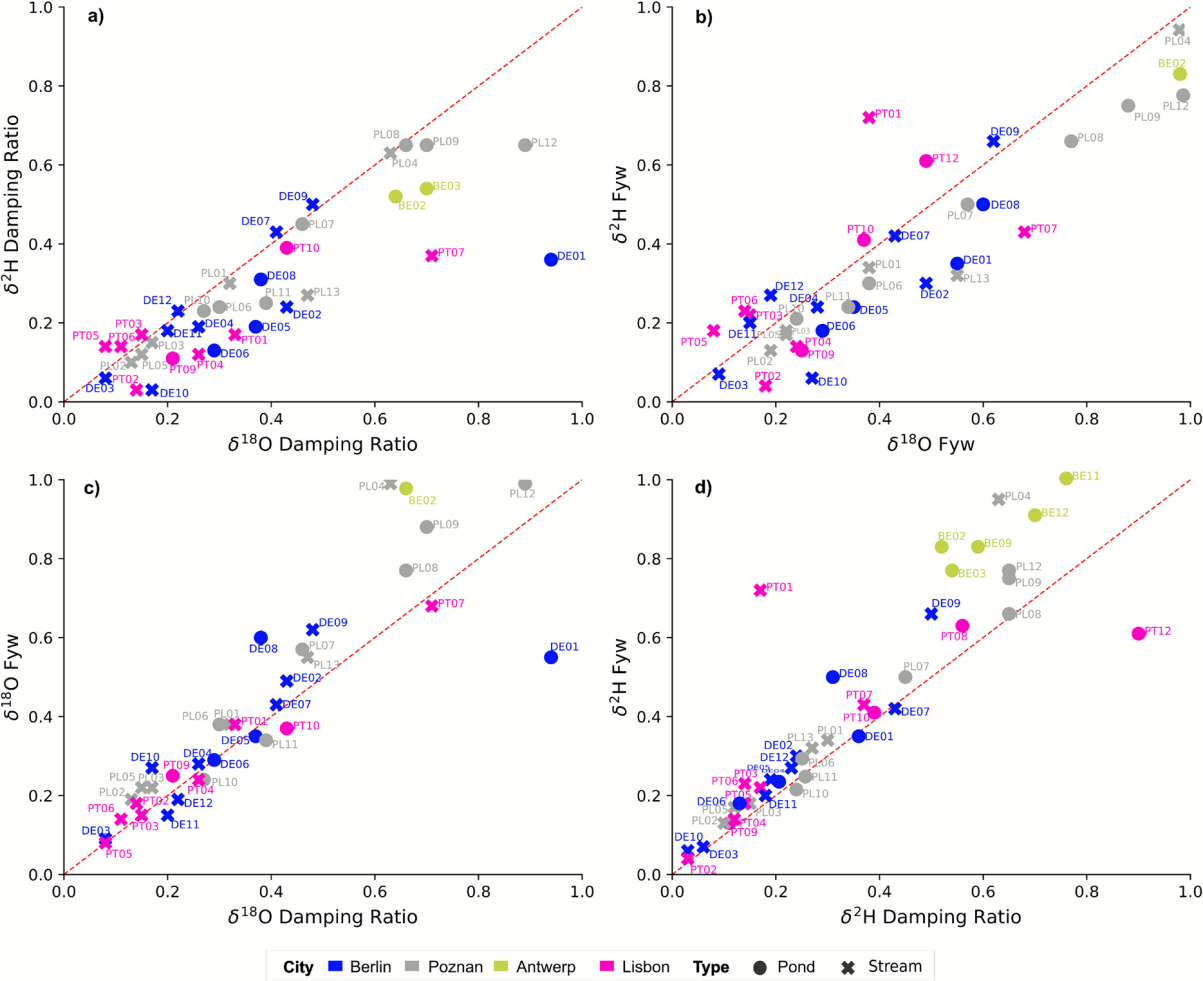
Mean transit time proxy measures with 1:1 line as red dashed line. (**a**) Damping ratio and (**b**) Fyw for all aquaNBS sites. (**c**) and (**d**) Comparison of water age metrics based on δ^18^O and δ^2^H data.

Despite coarse seasonal sampling, differences in the range of damping behavior and water ages were evident when comparing the different aquaNBS across all cities (Fig. 4). Low damping ratios (DR) (< 0.2) — meaning greater damping (i.e., less variability) of isotopic signals indicates limited rainfall-runoff responses and higher contributions from previously stored water. This was primarily observed in multiple stream locations in Lisbon, Poznań, and Berlin (Fig. 4a). In cases where potentially greater damping in δ^2^H signals, compared to δ^18^O, was observed, such as in two stream sites in Berlin (DE07, DE09) and some stream sites in Lisbon (PT03, PT05, PT06), this partially also suggests evaporative fractionation effects in these largely intermittent systems as well as more contributions from previously stored water, rather than direct contributions through precipitation or runoff. In contrast, higher DR (> 0.6), meaning less damped and more variable isotopic signatures due to direct rainfall-runoff responses, were mostly observed in ponds in Berlin, Antwerp, and Poznań and one stream location in Lisbon (PT07).

In terms of water ages, several streams in Berlin, Poznań, and Lisbon exhibited lower F_yw_ (< 0.2), indicating that in these systems older water sources (i.e., groundwater) likely dominate (Fig. 4b). Conversely in ponds, higher F_yw_ (> 0.8) could be observed, which signifies an overwhelming dominance of recent precipitation and thus stronger runoff responses, corresponding to the higher DR. In Poznań as well as Berlin, the average F_yw_ for ponds was higher than for streams (Berlin: ponds = 0.45, streams = 0.31; Poznań: ponds = 0.6, streams = 0.43). This indicates that in ponds, approximately 50% of the water originates from direct rainfall-runoff responses, making this a key water source to this type of aquaNBS. In contrast, the low DR and low F_yw_ in certain streams in Poznań (PL02, PL05, PL03) indicate that the majority of water in these systems is likely older than 3 months, stemming from previously stored water such as groundwater rather than direct precipitation or runoff.

In Lisbon, several F_yw_ estimates based on δ^2^H were much higher than δ^18^O, indicating potentially confounding effects of evaporative enrichment on δ^2^H and potential limitations for using these values when modeling transit times. δ^18^O F_yw_ in Lisbon was on average 0.26 in streams, whereas in ponds, F_yw_ was 0.55, indicating that the water in ponds likely consists of a mixture of recent and older water sources, whereas most stream water appears older than 3 months. Only stream sites exhibited a low DR and F_yw_ (both < 0.2), indicating potentially longer transit times in these aquaNBS sites and an influence of water sources > 3 months, with no discernible influence of evaporative fractionation (Fig. 4c, d). In comparison, in other sites with high DR and F_yw_ (PT12; pond) or low DR but high F_yw_ (PT01; stream), mixing of different water sources is more likely at these sites with more evaporation. Conversely, in Antwerp, F_yw_ estimates for ponds largely exceeded 100%, regardless of whether δ^2^H or δ^18^O was used, indicating that water in ponds consists almost 100% of water < 3 months old, which is in line with their primary function as rainwater retention pond (see Table S3 in the Supplementary Material for all Fyw and DR results).

**Fig. 5:**
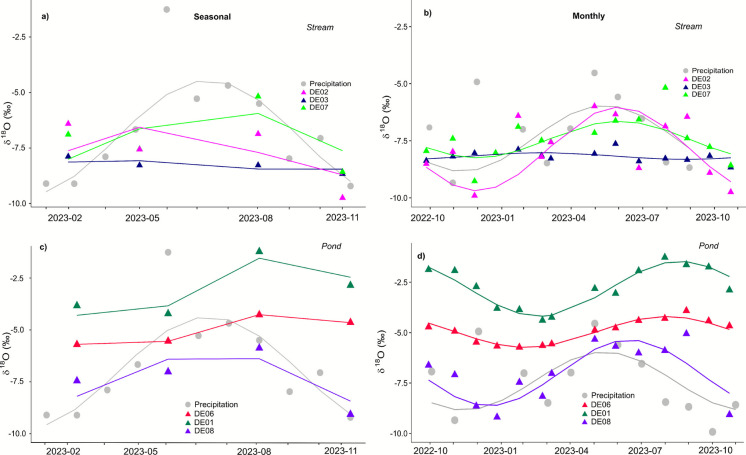
Comparison of young water fraction estimates using (**a**) and (**c**) seasonal or (**b**) and (**d**) monthly δ^18^O data for three stream sites (**a** and **b**) and (**c**) and three pond sites (**c** and **d**) in Berlin. Sinusoidal cycles were fitted to precipitation (grey) and seasonal or monthly isotopes from aquaNBS sites using iteratively weighted least squares regression (IRLS) for estimates of young water fractions (F_yw_).

A greater number of samples in Berlin allowed us to test the effect of sampling frequency. Comparing F_yw_ estimates from seasonally sampled data with monthly data for sites in Berlin, the effectiveness of transit time proxies to capture seasonal variations became apparent (Fig. 5). Except for one location (DE07), F_yw_ estimates of selected stream sites were broadly similar between seasonal and monthly sampled data (Fig. 5a, b). From a purely statistical standpoint, monthly data produced statistically significant sine-wave fits for the data (see Table 3). Nevertheless, the results indicate that even coarser seasonal data still allow meaningful characterizations of transit times, and general inference of transit time distributions. Especially in streams with low isotopic variability (i.e., DE03) due to consistent inflow of less isotopically variable water sources (such as treated wastewater effluent or groundwater), seasonal and monthly sampling revealed very similar results. Ponds showed some differences in F_yw_ estimates, with monthly data producing higher estimates of F_yw_ in all sites. As most ponds are strongly influenced by rainfall-runoff responses, the seasonality and damping of isotope signatures were significantly better captured by monthly sampling data (Fig. 5c, d) and could be even better characterized with higher frequency data (i.e., weekly or daily).

**Table 3:** Summary of F_yw_ estimates using seasonal and monthly δ^18^O data for streams (DE02, DE03, DE07) and ponds (DE01, DE06, DE08), including significance of model estimates (*p*-value), mean square error (R^2^) and adjusted R^2.^

Site	δ^18^O F_yw_		*p*-value		R^2^		R^2^_adj_			RMSE	
	*Seas*	*Mon*	*Seas*	*Mon*	*Seas*	*Mon*	*Seas*		*Mon*	*Seas*	*Mon*
DE02	0.49	0.55	0.75	0.001	0.42	0.68	− 0.72	0.63			
DE03	0.09	0.09	0.77	0.018	0.41	0.26	− 0.78	0.13			
DE07	0.43	⁓1.0	0.8	0.0002	0.35	0.78	− 0.94	0.74			
DE01	0.55	0.94	0.33	< 0.01	0.88	0.92	0.66	0.91		0.7	0.3
DE06	0.29	0.43	< 0.01	0.01	0.99	0.95	0.99	0.94		0.017	0.13
DE08	0.6	⁓1.0	0.54	< 0.01	0.7	0.65	0.1	0.59		1.21	0.88

## Discussion

### Implications of seasonal water source variability and residence times in urban aquatic NBS

Synoptic seasonal isotope sampling of different types of aquaNBS across a major hydroclimate gradient of four European cities revealed distinct water source and flowpath dynamics shaped by local hydroclimate conditions. Despite the interest in aquaNBS and their (often unknown) differences in design and size, the lack of a functional understanding of the hydrological processes can be aided through stable water isotope analysis to support implementation and management.

Most apparent were the differences between stream and pond-based aquaNBS, with ponds in Berlin, Antwerpen and Lisbon showing substantial seasonal differences between warm and cool season samples (Fig. 3). Due to the location in the dry NE of Germany, Berlin pond-based aquaNBS in particular, experienced significant evaporative enrichment. Similarly, the more enriched signatures in Antwerp in summer suggested a greater impact of hydroclimate in urban rainwater retention ponds during warm and dry periods there. A previous study of the same pond systems noted largely shallow groundwater levels in the area, which responded rapidly to precipitation as well as to dry periods (Martín Muñoz et al., 2023). Although these systems appear to be linked to groundwater, they still heavily depend on precipitation for recharge, particularly in such high rainfall/low energy (meaning low PET) environments. Conversely, the limited impact of evaporative enrichment across sites in Poznań indicates less seasonal variability of water sources in aquaNBS, due to more intensive summer precipitation and moderate temperatures, which likely buffer against seasonal extremes in this region.

Based on the seasonal patterns in water source dynamics and the importance of rainfall-runoff responses in urban pond-based aquaNBS observed in all cities, new questions emerge regarding the potential impacts of projected changes in the timing and delivery of precipitation. Greater variability in urban water source contributions (i.e. effluent discharge, storm drainage) in anthropogenically altered catchments has been linked to increasing hydrological variability (Marx et al., 2021; Strokal et al., 2021; Wild et al., 2024). This warrants consideration when designing aquaNBS concepts, to avoid undesired hydrological outcomes through either increasing flashiness or lack of water inflow. Especially during dry periods, urban rain water retention ponds may become stagnant, and without additional water inflow, turn eutrophic, with extensive and often harmful algae blooms, which poses a risk to water quality and public health (Grogan et al., 2023; Warter et al., 2024b, 2024c, 2024d).

For stream-based aquaNBS, projected trends of increasing river intermittency across Europe signal potential future limitations in water permanence, especially in low-rainfall/high-energy environments such as the Mediterranean (Stahl et al., 2010; Tramblay et al., 2021). Decreasing streamflow trends have already been observed in Spain (Coch & Mediero, 2016), Italy (De Girolamo et al., 2017), and Germany (Bormann & Pinter, 2017). Especially in southern and eastern regions of Europe, the number of zero-flow days is expected to increase and occur earlier in the season (Tramblay et al., 2021). Such conditions were already observed in Lisbon during the sampling period in early 2023, where a relatively dry first half of 2023 (Feb–Aug) with only ⁓60 mm of precipitation, resulted in multiple sample locations drying out earlier than expected. Increasing intermittency has also been observed in Berlin and many lowland regions of central and northern Europe, with profound implications to water quality, groundwater recharge and biogeochemical processes (Kleine et al., 2020; Smith et al., 2021; Wang et al., 2025; Wu et al., 2021; Ying et al., 2024). Arguably, intermittent river and ephemeral stream networks in the Mediterranean and other drought-prone regions are naturally adapted to cycles of flow cessation and dry periods (Datry et al., 2018). However, the projected rise in temperatures is likely to drive further changes in the frequency and length of intermittent periods (i.e., longer, earlier) as well as more frequent extended drought periods. This can affect initial conditions of intermittent streams beyond the usual drying or low flow conditions (Sarremejane et al., 2022). Such climate-induced changes to streamflow permanence may induce widespread permanent flow regime shifts from perennial to intermittent, and even potentially cross irreversible thresholds beyond usual drying conditions, challenging the functionality of aquaNBS (Döll & Schmied, 2012; Sarremejane et al., 2022).

In this way, current flow regimes and potential abiotic implications of climate-driven changes in streamflow permanence must be considered when developing aquaNBS under projected climate conditions. In addition, local characteristics, such as size and structure of any aquaNBS, underlying geology, surrounding land cover, connectedness and main water sources have significant implications for flow conditions. Our stable isotope results highlight how different features store and route local runoff and benefit from groundwater supplementation or other urban water sources differently. In Berlin, baseflows of many streams are supplemented through a significant portion of treated effluent discharge, artifically raising flows to pre-empt zero-flow conditions (Marx et al., 2021, 2023). Stream-based aquaNBS that receive such treated effluent discharge may be more buffered against drought periods, as treated wastewater discharge is modulated to maintain elevated baseflows (Kuhlemann et al., 2020; Marx et al., 2021; Warter et al., 2024a) during dry periods, when most water is consumed by evapotranspiration (Smith et al., 2021). However, the augmentation of streamflow with recycled wastewater faces its own unique hydrological challenges with potential ecological limitations, which warrant serious consideration when implementing aquaNBS in such systems (Büttner et al., 2022; Plumlee et al., 2012; Wild et al., 2024).

### ,Differences in water residence times across urban aquaNBS

As part of this study, we hypothesized that TTP proxies using seasonal isotope data can be powerful enough to characterize the hydrologic functioning of different aquaNBS across Europe. Typically, in urban isotope studies, higher-resolution data (e.g., monthly, fortnightly) are preferred when trying to identify dominant controls on hydrological processes (Bonneau et al., 2018; Stevenson et al., 2022; Von Freyberg et al., 2022; Warter et al., 2024b). Thus, when sampling frequency is fairly coarse (e.g., seasonal) due to logistical or financial constraints, which are common in urban settings, a question pertains as to how this affects estimations of residence times. Based on our results, seasonal TTP metrics were effective in revealing a clear dichotomy in water storage and residence, also seen in the actual isotopic signatures, between pond-based and stream-based aquaNBS. The isotope-based diagnostics indicated that ponds and streams exhibited distinctly different fractions of recent and older water, but did not indicate that this would hold true across any hydroclimate gradient. A caveat to consider is that further flow-controlloing factors indeed include construction and connectedness of the pond or stream channel, which would also affect water storage and residence times. Still, isotope-tracers showed how the different aquaNBS features stored and routed runoff and groundwater supplementation, with pond-based aquaNBS showing a greater influence of seasonal precipitation and rainfall-runoff responses, while stream-based aquaNBS exhibited overall slower, more groundwater-influenced dynamics and mixing behavior.

Considering future warming and drying trends across western (Lima et al., 2023) and central Europe (Larsen et al., 2020), projected changes to local water balances (i.e., increase in PET, decrease in precipitation) will likely affect the hydrological functioning of water-based aquaNBS. Given the complexity of the urban water cycle and the fact that hydrologic responses may not be uniform across catchments, we argue that using isotope-based diagnostics are very insightful for attempting to differentiate between slow and fast responding systems, how water is stored and routed in different features, and what kind of water sources (i.e., groundwater, precipitation, effluent) are contributing to stream or pond-based aquaNBS features. However, to determine how exactly the aquaNBS features might respond to different hydroclimate conditions, future projections of how climate may change the water balance will need to be considered.

**Fig. 6:**
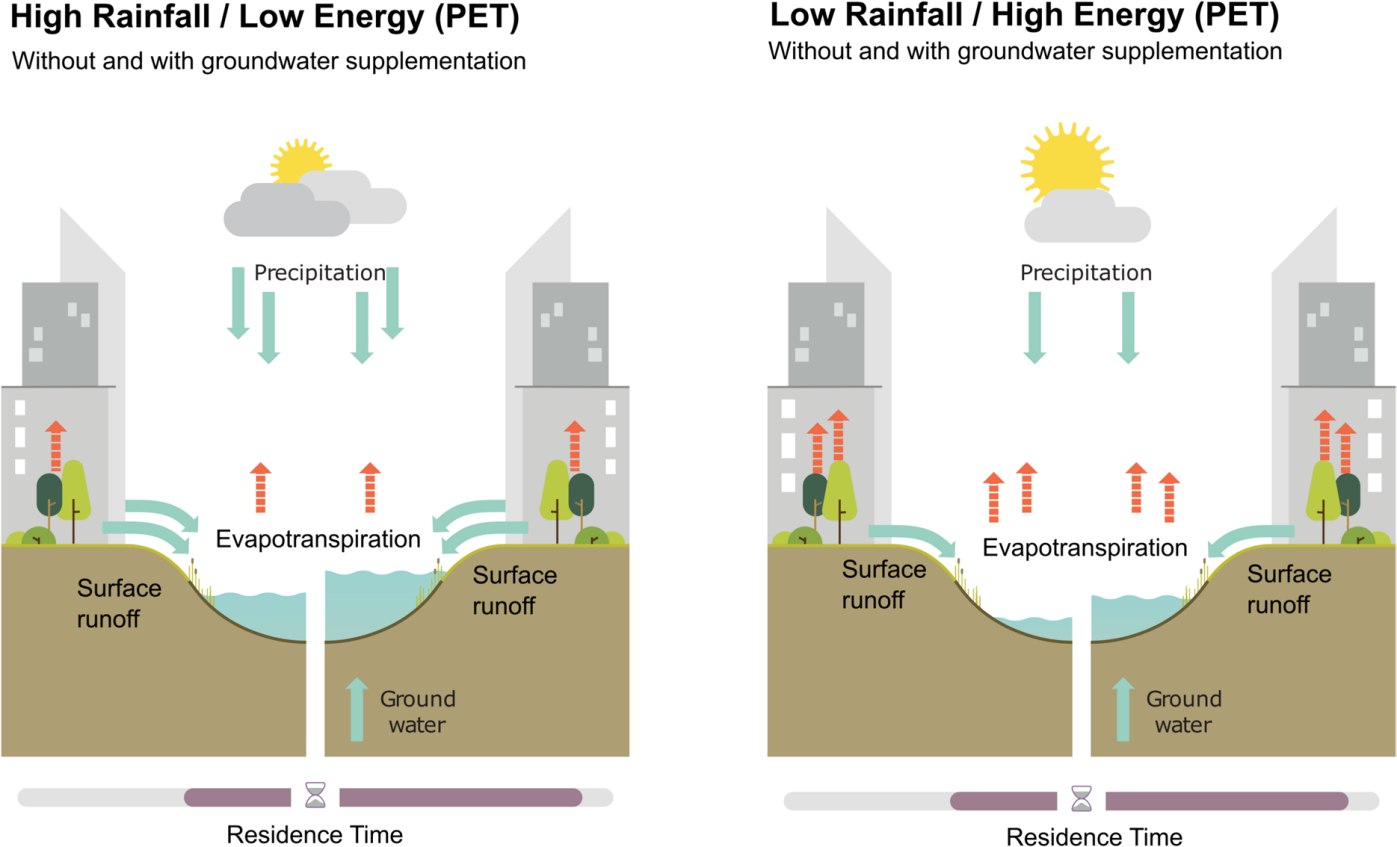
Schematic representation illustrating the influence of urban water sources on residence time responses in different urban aquaNBS in high rainall/low energy (left) and low rainfall/high energy (right) environments.

Based on the overall higher F_yw_ in ponds (mean 0.65), we can assume a stronger impact of recent rainfall and urban runoff responses. Especially in high rainfall/low energy environments like Antwerp (Fig. 6a), recent water inputs where dominant, as indicated by the higher F_yw_ (> 0.9). There, a certain homogeneity in runoff processes suggests shorter transit time dynamics, as would be expected of rapidly responding rainwater retention ponds that quickly capture and store excess water during extreme precipitation events. At the same time, during dry periods, the lack of water inflow and dilution as well as increased evaporative loss can potentially develop into adverse conditions with widespread implications for water quality and nutrient retention in such aquaNBS. As a result, elevated thermal and chemical stress during extreme events (i.e., flood or drought) — either through increased pollution inflow or lack of dilution through an additional water source, such as groundwater — represents a risk to such single-source pond systems. This can limit their ecological potential due to rapid development of toxic algal blooms and more eutrophic conditions due to water pollution (Grogan et al., 2023; Oertli & Parris, 2019; Warter et al., 2024b, 2024c, 2024d).

Across all stream-based aquaNBS, lower F_yw_ (mean: 0.3) implied the presence of slower subsurface flow processes and local groundwater influence. One caveat to this conclusion is the overwhelming dominance of treated effluent discharge in some streams in Berlin, which results in extreme damping of the isotope signature and very low F_yw_ estimates (Marx et al., 2021; Warter et al., 2024b, 2024c, 2024d). Generally, it can be said that subcatchments with larger F_yw_ tend to have predominately shorter transit times due to more rapid rainfall-runoff responses, thus responding also more rapidly to solute input in streamflow and lower nutrient retention capacities (Lutz et al., 2018), as seen in pond-based systems in Antwerp. At the same time, in catchments with lower F_yw_, other subsurface processes are likely contributing to streamflow (i.e., groundwater), signaling less dependence on rainfall to sustain flow/water levels and a greater resilience to hydroclimate variability and streamflow intermittence.

The differences observed underscore the need to match NBS design to local hydrological and climate conditions, such as rainfall intensity and drought frequency, as well as urban modifications like soil compaction, reduced vegetative cover, and catchment fragmentation (Gessner et al., 2014; Oswald et al., 2023). Thus, when assessing water requirements of different aquaNBS, TTP proxies provide valuable insights into inter- and intra-geographical differences in water balance dynamics and nutrient retention. This study, has shown that even at a low frequency, isotope data can yield sufficient hydrological context, which provides a possible explanation for heterogeneities in hydrological function across water bodies with diverse water sources and transit time (Jung et al., 2025). Hence, we argue that there is great value in pursuing stable isotope assessments in the context of evaluating and monitoring aquaNBS at the urban catchment scale, to better establish hydrological boundary conditions, assess any potential limitations in terms of seasonal water availability or varying urban water source contributions. In future, decision makers should be guided, in part, by evidence derived from such isotope-based diagnostics.

### Implications for future urban aquaNBS

Our findings highlight how the critical need for monitoring of blue and green infrastructure could be addressed. At the same time, it requires future projections to determine how aquaNBS features would change and how changes in the water balance would affect hydrologic functioning. In general, projected trends of increased summer drought and precipitation deficits in high rainfall/low energy (low PET) environments such as Belgium (Tabari et al., 2015) have the potential to negatively affect pond-based aquaNBS, through increased water loss, particularly in systems where no groundwater supplementation occurs (Fig. 6a). Similarly, water-based aquaNBS in low rainfall/high energy (high PET) environments such as Lisbon, may be decreasing in their functionality as magnitude, frequency and dynamics of rainfall, temperature and runoff might be altered in future (Fig. 6b) (Lima et al., 2023). In both cases, groundwater supplementation plays an important role in buffering any negative effects of drought, while single-source systems (i.e., only surface runoff), are likely more exposed to evaporative losses and ecologic deterioration. That said, under severe dry conditions, the lack of total available water for runoff, soil water storage and susbeqeuntly groundwater recharge may have detrimental consequences across all ecosystems.

Already, the 2018–2023 drought, characterized by extreme heat and severe precipitation deficits, severely impacted water resources throughout central Europe (Hari et al., 2020; Rousi et al., 2023). Especially in groundwater-dependent cities like Berlin, blue and green infrastructure suffered from severe water deficits and declining groundwater levels, calling into question future urban water management and the importance of climate-sensitive water resource management that supports blue and green infrastrucuture (Pohle et al., 2025).

The results of this study underscore the value of a more integrated, hydrologically informed approach to the planning and implementation of urban aquaNBS, by using reconnaissance-style surveys of stable water isotopes. In densely urbanized areas, the implementation of NBS tends to occur in a piecemeal site-specific way, based on engineering design (e.g., to accommodate runoff from rainfall of a particular return period), with limited cognisance of the local hydrological context, relevant water balance components (e.g., Miles & Band, 2015) and an understanding of cummulative effects on flow dynamics (Golden & Hoghooghi, 2018). In heavily transformed and managed urban catchments, such as the Panke catchment in Berlin or the Warta River catchment in Poznań, water balance dynamics throughout the catchment and its tributaries are invariably closely linked to surrounding land use and water management practices (Marx et al., 2023; Sojka, 2022). As humans alter flow regimes and runoff patterns in urban catchments through various land use changes and extraction of surface and groundwater, this raises the question whether aquaNBS are able to adjust to changes in the water balance in response to progressive global change or different water management practices (Krauze & Wagner, 2019).

Especially smaller, independent NBS, such as ponds or micro-reservoirs, are often designed to fit only local conditions and support sustainable water management or provide other social or aestethic ecosystem services. As a result, these anthropocentric approaches are prone to degradation or failure if abandoned and unmanaged or poorly integrated with the landscape.This can potentially increase the chances of ecosystem disservices and minimize any long-term success of nature-based solution approaches (Lalonde et al., 2024). Similarly, restoration of dried-out wetlands or reconnection of floodplains as a form of aquaNBS is a challenging task that requires consideration of catchment scale processes and potential stressors to avoid ecosystem disservices and the destabilization of the hydrological system (Lalonde et al., 2024). Especially considering hydroclimate extremes, enhancement of surface storage, reduction of runoff and urban cooling effects remain desired key functionalities of water-based NBS, which require a climate-conscious implementation.

As climate variability and anthropogenic pressures intensify, the effectivness and delivery of hydrological and ecological ecosystem services will increasingly depend on their alignment with local water source dynamics and hydroclimate regimes. Understanding whether a system is primarily rainfall-driven, responds strongly to rainfall-runoff processes, is groundwater-fed or reliant on anthropogenic inflows such as wastewater discharge, will define the performance and resilience of aquaNBS. This may be particularly relevant in lower income countries of the Global South, where urban water infrastructure often exists in close proximity to settlements and a high dependency on local ecosystems for basic needs and livelihoods puts significant pressure on limited water resources (Foster et al., 2020; Ogidi, 2024). In such settings, aquaNBS can help to reduce water risks to economies and society (Acreman et al., 2021).

Although aquaNBS are often viewed as ready-to-use units to be implemented in specific locations to address local problems, it is crucial to obtain a fundamental hydrologic process understanding into NBS design approaches (Pinho et al., 2023). In this context, evaluating the implications of different residence times and hydroclimate variability through stable isotopes and TTPs has great value for assessing relevant water partitioning processes and flowpaths in ungauged urban hydrosystems. Further research might explore also explicit linkages to surrounding land use and biodiversity metrics (i.e., eDNA, macrophyte diversity, macrointertebrates) to evaluate the impact of hydrology on ecological ecosystem services – an aspect that is still underappreciated in the context of NBS monitoring (Szoszkiewicz et al., 2024; Warter et al., 2024b, 2024c, 2024d).

## Conclusion

The successful implementation of water-related nature-based solutions (aquaNBS) for urban water management requires consideration of the underlying hydrological processes and hydroclimate interactions within a catchment context. In this study, we showed that through seasonal sampling of stable water isotopes s, the main water sources and flow paths within different types of aquaNBS could be well characterized across a major hydroclimate gradient. The application of transit time proxies, such as tracer damping and young water fraction estimations, has shown that ponds were potentially more sensitive to hydroclimate changes, as evidenced by the strong seasonality in evaporative enrichment and high fractions of young water contributions. In contrast, most streams indicated greater mixing of water sources and longer transit times, suggesting potentially greater resilience to hydroclimate variability. In addition, a comparison between seasonally sampled data and monthly sampling for selected locations in Berlin showed that even relatively coarse temporal data on a seasonal basis, but with more extensive spatial coverage, can be insightful for broader hydrologic characterizations of aquaNBS at larger scales, and provide crucial understanding of local hydrological processes and hydroclimate sensitivities. This has further implications for evaluating risks to water quality, urban water management and various ecological and social ecosystem services. The study fills an important knowledge gap regarding the fundamental understanding of aquaNBS and highlights the potential to use stable water isotopes as a monitoring framework to support the design and implementation of urban aquaNBS.

## Supplementary Information

Below is the link to the electronic supplementary material.

ESM 1 (DOCX 29.5 KB)

## Data Availability

Stable water isotope data will be available on request. Metadata is stored on the IGB Freshwater Research and Environmental Database (FRED) under 10.18728/igb-fred-984.0.

## References

[CR1] Acreman, M., Smith, A., Charters, L., Tickner, D., Opperman, J., Acreman, S., Edwards, F., Sayers, P., & Chivava, F. (2021). Evidence for the effectiveness of nature-based solutions to water issues in Africa. *Environmental Research Letters*. 10.1088/1748-9326/ac0210

[CR2] Benettin, P., Volkmann, T. H. M., Von Freyberg, J., Frentress, J., Penna, D., Dawson, T. E., & Kirchner, J. W. (2018). Effects of climatic seasonality on the isotopic composition of evaporating soil waters. *Hydrology and Earth System Sciences,**22*(5), 2881–2890. 10.5194/hess-22-2881-2018

[CR3] Bhuiyan, S. A., Jameel, Y., Chartrand, M. M. G., St-Jean, G., Gibson, J., & Bataille, C. P. (2023). Spatial variations in tap water isotopes across Canada: Tracing water from precipitation to distribution and assess regional water resources. *PLOS Water,**2*(1), Article e0000068. 10.1371/journal.pwat.0000068

[CR4] Bochenek, D., Gorzkowska, E., Hejne, J., Kafara, E., Kielczykowska, A., Marciniak, K., Nowakowska, B., Siewara, W., Wronski, M., & Wrzosek, A. (2024). *Ochrona środowiska 2024 - Environment 2024*. Statistics Poland, Agriculture and Environment Department, ISSN 0867–3217

[CR5] Bonneau, J., Burns, M. J., Fletcher, T. D., Witt, R., Drysdale, R. N., & Costelloe, J. F. (2018). The impact of urbanization on subsurface flow paths – A paired-catchment isotopic study. *Journal of Hydrology,**561*(January), 413–426. 10.1016/j.jhydrol.2018.04.022

[CR6] Bormann, H., & Pinter, N. (2017). Trends in low flows of German rivers since 1950: Comparability of different low-flow indicators and their spatial patterns. *River Research and Applications,**33*(7), 1191–1204. 10.1002/rra.3152

[CR7] Bowen, G. J. (2025). *OIPC: The Online Isotopes in Precipitation Calculator, version 3.1*. http://www.waterisotopes.org.

[CR8] Burns, M. J., Fletcher, T. D., Walsh, C. J., Ladson, A. R., & Hatt, B. E. (2012). Hydrologic shortcomings of conventional urban stormwater management and opportunities for reform. *Landscape and Urban Planning,**105*(3), 230–240. 10.1016/j.landurbplan.2011.12.012

[CR9] Bush, J., & Doyon, A. (2019). Building urban resilience with nature-based solutions: How can urban planning contribute? *Cities,**95*(September), Article 102483. 10.1016/j.cities.2019.102483

[CR10] Büttner, O., Jawitz, J. W., Birk, S., & Borchardt, D. (2022). Why wastewater treatment fails to protect stream ecosystems in Europe. *Water Research,**217*(March), Article 118382. 10.1016/j.watres.2022.118382 35413560 10.1016/j.watres.2022.118382

[CR11] Chowdhury, K., Basu, S., Pramanik, M., & Plieninger, T. (2025). Blue infrastructure as nature-based solutions for urban sustainability: Evaluating local perceptions from four Indian megacities. *Nature-Based Solutions*. 10.1016/j.nbsj.2025.100211

[CR12] Coch, A., & Mediero, L. (2016). Trends in low flows in Spain in the period 1949–2009. *Hydrological Sciences Journal,**61*(3), 568–584. 10.1080/02626667.2015.1081202

[CR13] Craig, H., Gordon, L. I., & Horibe, Y. (1963). Isotopic exchange effects in the evaporation of water: 1 Low-temperature experimental results. *Journal of Geophysical Research (1896-1977),**68*(17), 5079–5087. 10.1029/JZ068i017p05079

[CR14] Dansgaard, W. (1964). Stable isotopes in precipitation. *Tellus,**16*(4), 436–468. 10.3402/tellusa.v16i4.8993

[CR15] Datry, T., Boulton, A. J., Bonada, N., Fritz, K., Leigh, C., Sauquet, E., Tockner, K., Hugueny, B., & Dahm, C. N. (2018). Flow intermittence and ecosystem services in rivers of the Anthropocene. *Journal of Applied Ecology,**55*(1), 353–364. 10.1111/1365-2664.12941 29681651 10.1111/1365-2664.12941 PMC5907507

[CR16] Davis, M., & Naumann, S. (2017). *Making the case for sustainable urban drainage systems as a nature-based solution to urban flooding*. 10.1007/978-3-319-56091-5_8

[CR17] De Girolamo, A. M., Bouraoui, F., Buffagni, A., Pappagallo, G., & Lo Porto, A. (2017). Hydrology under climate change in a temporary river system: Potential impact on water balance and flow regime. *River Research and Applications,**33*(7), 1219–1232. 10.1002/rra.3165

[CR18] Döll, P., & Schmied, H. M. (2012). How is the impact of climate change on river flow regimes related to the impact on mean annual runoff? A global-scale analysis. *Environmental Research Letters*. 10.1088/1748-9326/7/1/014037

[CR19] DWD, Deutscher Wetter Dienst (2023). *Vieljährige Mittelwerte Deutschland/Longterm Climate Means for Germany*. https://www.dwd.de/DE/leistungen/klimadatendeutschland/vielj_mittelwerte.html

[CR20] DWD, Deutscher Wetter Dienst (2024). *Climate Data Center (CDC)*. https://opendata.dwd.de/climate_environment/CDC/

[CR21] Ehleringer, J. R., Barnette, J. E., Jameel, Y., Tipple, B. J., Bowen, J., Ehleringer, J. R., Barnette, J. E., Jameel, Y., & Tipple, B. J. (2016). Urban water – A new frontier in isotope hydrology. *Isotopes in Environmental and Health Studies*. 10.1080/10256016.2016.1171217 10.1080/10256016.2016.1171217 27142528

[CR22] Evaristo, J., Jasechko, S., & McDonnell, J. J. (2015). Global separation of plant transpiration from groundwater and streamflow. *Nature,**525*(7567), 91–94. 10.1038/nature14983 26333467 10.1038/nature14983

[CR23] Everard, M., & Moggridge, H. L. (2012). Rediscovering the value of urban rivers. *Urban Ecosystems,**15*(2), 293–314. 10.1007/s11252-011-0174-7

[CR24] Foster, S., Eichholz, M., Nlend, B., & Gathu, J. (2020). Securing the critical role of groundwater for the resilient water-supply of urban Africa. *Water Policy,**22*(1), 121–132. 10.2166/wp.2020.177

[CR25] Gessner, M. O., Hinkelmann, R., Nützmann, G., Jekel, M., Singer, G., Lewandowski, J., Nehls, T., & Barjenbruch, M. (2014). Urban water interfaces. *Journal of Hydrology,**514*, 226–232. 10.1016/j.jhydrol.2014.04.021

[CR26] Golden, H. E., & Hoghooghi, N. (2018). Green infrastructure and its catchment-scale effects: An emerging science. *Wiley Interdisciplinary Reviews: Water,**5*(1), 1–14. 10.1002/WAT2.1254 10.1002/wat2.1254 PMC5907509 29682288

[CR27] Grogan, A. E., Alves-de-Souza, C., Cahoon, L. B., & Mallin, M. A. (2023). Harmful algal blooms: A prolific issue in urban stormwater ponds. *Water*. 10.3390/w15132436

[CR28] Hack, J., & Schröter, B. (2020). Nature-based solutions for river restoration in metropolitan areas. *The Palgrave Encyclopedia of Urban and Regional Futures*. 10.1007/978-3-030-51812-7

[CR29] Hari, V., Rakovec, O., Markonis, Y., Hanel, M., & Kumar, R. (2020). Increased future occurrences of the exceptional 2018–2019 Central European drought under global warming. *Scientific Reports,**10*(1), 1–10. 10.1038/s41598-020-68872-9 32764540 10.1038/s41598-020-68872-9 PMC7413549

[CR30] Hrachowitz, M., Soulsby, C., Tetzlaff, D., Malcolm, I. A., & Schoups, G. (2010). Gamma distribution models for transit time estimation in catchments: Physical interpretation of parameters and implications for time-variant transit time assessment. *Water Resources Research*. 10.1029/2010WR009148

[CR31] IAEA & WMO (2024). *Global Network of Isotopes in Precipitation. The GNIP Database*. https://nucleus.iaea.org/wiser

[CR32] IMGW-PIB. (2024). *Danepubliczne*. https://danepubliczne.imgw.pl/

[CR33] Jameel, Y., Brewer, S., Fiorella, R. P., Tipple, B. J., Terry, S., & Bowen, G. J. (2018). Isotopic reconnaissance of urban water supply system dynamics. *Hydrology and Earth System Sciences,**22*(11), 6109–6125. 10.5194/hess-22-6109-2018

[CR34] Jefferson, A. J., Bell, C. D., Clinton, S. M., & Mcmillan, S. K. (2015). Application of isotope hydrograph separation to understand contributions of stormwater control measures to urban headwater streams. *Hydrological Processes,**29*(25), 5290–5306. 10.1002/hyp.10680

[CR35] Jung, H., Tetzlaff, D., Birkel, C., & Soulsby, C. (2025). Recent developments and emerging challenges in tracer-aided modeling. *Wires Water*. 10.1002/wat2.70015

[CR36] Kabisch, N., Frantzeskaki, N., Pauleit, S., Naumann, S., Davis, M., Artmann, M., Haase, D., Knapp, S., Korn, H., Stadler, J., Zaunberger, K., & Bonn, A. (2016). Nature-based solutions to climate change mitigation and adaptation in urban areas: Perspectives on indicators, knowledge gaps, barriers, and opportunities for action. *Ecology and Society*. 10.5751/ES-08373-210239

[CR37] Kendall, C., & McDonnell, J. J. (1998). *Isotope Tracers in Catchment Hydrology*. Elsevier Science. 10.1016/C2009-0-10239-8

[CR38] Kirchner, J. W. (2016). Aggregation in environmental systems-Part 1: Seasonal tracer cycles quantify young water fractions, but not mean transit times, in spatially heterogeneous catchments. *Hydrology and Earth System Sciences,**20*(1), 279–297. 10.5194/hess-20-279-2016

[CR39] Kleine, L., Tetzlaff, D., Smith, A., Wang, H., & Soulsby, C. (2020). Using water stable isotopes to understand evaporation, moisture stress, and re-wetting in catchment forest and grassland soils of the summer drought of 2018. *Hydrology and Earth System Sciences,**24*(7), 3737–3752. 10.5194/hess-24-3737-2020

[CR40] Krauze, K., & Wagner, I. (2019). From classical water-ecosystem theories to nature-based solutions — Contextualizing nature-based solutions for sustainable city. *Science of the Total Environment,**655*, 697–706. 10.1016/j.scitotenv.2018.11.187 30476850 10.1016/j.scitotenv.2018.11.187

[CR41] Krueger, E. H., Borchardt, D., Jawitz, J. W., & Rao, P. S. C. (2020). Balancing security, resilience, and sustainability of urban water supply systems in a desirable operating space. *Environmental Research Letters*. 10.1088/1748-9326/ab6c2d

[CR42] Kuhlemann, L. M., Tetzlaff, D., & Soulsby, C. (2020). Urban water systems under climate stress: An isotopic perspective from Berlin, Germany. *Hydrological Processes,**34*(18), 3758–3776. 10.1002/hyp.13850

[CR43] Kuhlemann, L. M., Tetzlaff, D., & Soulsby, C. (2021). Spatio-temporal variations in stable isotopes in peri-urban catchments: A preliminary assessment of potential and challenges in assessing streamflow sources. *Journal of Hydrology,**600*(July), 126685. 10.1016/j.jhydrol.2021.126685

[CR44] Lalonde, M., Drenkhan, F., Rau, P., Baiker, J. R., & Buytaert, W. (2024). Scientific evidence of the hydrological impacts of nature-based solutions at the catchment scale. *Wiley Interdisciplinary Reviews: Water*, *February 2023*, 1–19. 10.1002/wat2.1744

[CR45] Lammers, R. W., Dell, T. A., & Bledsoe, B. P. (2020). Integrating stormwater management and stream restoration strategies for greater water quality benefits. *Journal of Environmental Quality,**49*(3), 569–581. 10.1002/jeq2.20047 33016400 10.1002/jeq2.20047

[CR46] Landwehr, J. M., & Coplen, T. (2006). Line-conditioned excess: A new method for characterizing stable hydrogen and oxygen isotope ratios in hydrologic systems (IAEA-CN-118/56). In *Isotopes in Environmental Studies* (IAEA-CSP-2, Issue October, pp. 132–135). IAEA.

[CR47] Larsen, M. A. D., Petrović, S., Radoszynski, A. M., McKenna, R., & Balyk, O. (2020). Climate change impacts on trends and extremes in future heating and cooling demands over Europe. *Energy and Buildings*. 10.1016/j.enbuild.2020.110397

[CR48] Li, C., Sun, G., Caldwell, P. V., Cohen, E., Fang, Y., Zhang, Y., Oudin, L., Sanchez, G. M., & Meentemeyer, R. K. (2020). Impacts of urbanization on watershed water balances across the conterminous United States. *Water Resources Research*. 10.1029/2019WR026574

[CR49] Lima, D. C. A., Bento, V. A., Lemos, G., Nogueira, M., & Soares, P. M. M. (2023). A multi-variable constrained ensemble of regional climate projections under multi-scenarios for Portugal – Part II: Sectoral climate indices. *Climate Services,**30*, Article 100377. 10.1016/j.cliser.2023.100377

[CR50] Lutz, S. R., Krieg, R., Müller, C., Zink, M., Knöller, K., Samaniego, L., & Merz, R. (2018). Spatial patterns of water age: Using young water fractions to improve the characterization of transit times in contrasting catchments. *Water Resources Research,**54*(7), 4767–4784. 10.1029/2017WR022216

[CR51] Martín Muñoz, S., Schoelynck, J., Tetzlaff, D., Debbaut, R., Warter, M., & Staes, J. (2023). Assessing biodiversity and regulatory ecosystem services in urban water bodies which serve as aqua-nature-based solutions. *Frontiers in Environmental Science,**11*(January), 1–14. 10.3389/fenvs.2023.1304347

[CR52] Marx, C., Tetzlaff, D., Hinkelmann, R., & Soulsby, C. (2021). Isotope hydrology and water sources in a heavily urbanized stream. *Hydrological Processes,**35*(10), 1–20. 10.1002/hyp.14377

[CR53] Marx, C., Tetzlaff, D., Hinkelmann, R., & Soulsby, C. (2023). Effects of 66 years of water management and hydroclimatic change on the urban hydrology and water quality of the Panke catchment, Berlin. *Germany. Science of the Total Environment,**900*(July), 165764. 10.1016/j.scitotenv.2023.165764 37516173 10.1016/j.scitotenv.2023.165764

[CR54] Miles, B., & Band, L. E. (2015). Green infrastructure stormwater management at the watershed scale: Urban variable source area and watershed capacitance. *Hydrological Processes,**29*(9), 2268–2274. 10.1002/hyp.10448

[CR55] Morales, K., & Oswald, C. (2020). Water age in stormwater management ponds and stormwater management pond-treated catchments. *Hydrological Processes,**34*(8), 1854–1867. 10.1002/hyp.13697

[CR56] Numberger, D., Zoccarato, L., Woodhouse, J., Ganzert, L., Sauer, S., Márquez, J. R. G., Domisch, S., Grossart, H. P., & Greenwood, A. D. (2022). Urbanization promotes specific bacteria in freshwater microbiomes including potential pathogens. *Science of the Total Environment*, *845*(July). 10.1016/j.scitotenv.2022.157321 10.1016/j.scitotenv.2022.157321 35839872

[CR57] Oertli, B., & Parris, K. M. (2019). Review: Toward management of urban ponds for freshwater biodiversity. *Ecosphere*. 10.1002/ecs2.2810

[CR58] Ogidi, O. I. (2024). Strategies of sustainable management of water resources in the global south. In S. C. Izah, M. C. Ogwu, A. Loukas, & H. Hamidifar (Eds.), *Water Crises and Sustainable Management in the Global South* (pp. 391–422). Springer Nature Singapore. 10.1007/978-981-97-4966-9_13

[CR59] Oswald, C. J., Kelleher, C., Ledford, S. H., Hopkins, K. G., Sytsma, A., Tetzlaff, D., Toran, L., & Voter, C. (2023). Integrating urban water fluxes and moving beyond impervious surface cover: A review. *Journal of Hydrology,**618*, Article 129188. 10.1016/j.jhydrol.2023.129188

[CR60] Pinho, P., Haase, D., Gebler, D., Staes, J., Martelo, J., Schoelynck, J., Szoszkiewicz, K., Monaghan, M. T., & Vierikko, K. (2023). *Urban aquatic nature-based solutions in the context of global change: Uncovering the social-ecological-technological framework*. 139–157. 10.1007/978-3-031-34378-0_8

[CR61] Plumlee, M. H., Gurr, C. J., & Reinhard, M. (2012). Recycled water for stream flow augmentation: Benefits, challenges, and the presence of wastewater-derived organic compounds. *Science of the Total Environment*, *438*(October 2012), 541–548. 10.1016/j.scitotenv.2012.08.062 10.1016/j.scitotenv.2012.08.062 23041295

[CR62] Pohle, I., Zeilfelder, S., Birner, J., & Creutzfeldt, B. (2025). The 2018–2023 drought in Berlin: Impacts and analysis of the perspective of water resources management. *Natural Hazards and Earth System Sciences,**25*(4), 1293–1313. 10.5194/nhess-25-1293-2025

[CR63] Ress, L. D., & James, L. A. (2020). *Impacts of urban drainage systems on stormwater hydrology : Rocky Branch Watershed , Columbia , South Carolina*. *November 2019*, 1–13. 10.1111/jfr3.12643

[CR64] Richardson, M., & Soloviev, M. (2021). The urban river syndrome: Achieving sustainability against a backdrop of accelerating change. *International Journal of Environmental Research and Public Health*. 10.3390/ijerph18126406 10.3390/ijerph18126406 PMC8296234 34199215

[CR65] Ring, A. M., Tetzlaff, D., Dubbert, M., Freymueller, J., & Soulsby, C. (2024). Assessing the impact of drought on water cycling in urban trees via in-situ isotopic monitoring of plant xylem water. *Journal of Hydrology,**633*(April), 131020. 10.1016/j.jhydrol.2024.131020

[CR66] Rousi, E., Fink, A. H., Andersen, L. S., Becker, F. N., Beobide-Arsuaga, G., Breil, M., Cozzi, G., Heinke, J., Jach, L., Niermann, D., Petrovic, D., Richling, A., Riebold, J., Steidl, S., Suarez-Gutierrez, L., Tradowsky, J. S., Coumou, D., Düsterhus, A., Ellsäßer, F., & Xoplaki, E. (2023). The extremely hot and dry 2018 summer in central and northern Europe from a multi-faceted weather and climate perspective. *Natural Hazards and Earth System Sciences,**23*(5), 1699–1718. 10.5194/nhess-23-1699-2023

[CR67] Sarremejane, R., Messager, M. L., & Datry, T. (2022). Drought in intermittent river and ephemeral stream networks. *Ecohydrology*. 10.1002/eco.2390

[CR68] Smith, A., Tetzlaff, D., Kleine, L., Maneta, M., & Soulsby, C. (2021). Quantifying the effects of land use and model scale on water partitioning and water ages using tracer-aided ecohydrological models. *Hydrology and Earth System Sciences,**25*, 2239–2259. 10.5194/hess-25-2239-2021

[CR69] SNIRH. (2024). *Sistema Nacional de Informação - de Recursos Hidricos*. https://snirh.apambiente.pt/index.php?idMain=2&idItem=1&objCover=920123704&objSite=920685506

[CR70] Sojka, M. (2022). Directions and extent of flows changes in Warta River Basin (Poland) in the context of the efficiency of run-of-river hydropower plants and the perspectives for their future development. *Energies*. 10.3390/en15020439

[CR71] Soulsby, C., & Tetzlaff, D. (2008). Towards simple approaches for mean residence time estimation in ungauged basins using tracers and soil distributions. *Journal of Hydrology,**363*(1–4), 60–74. 10.1016/j.jhydrol.2008.10.001

[CR72] Soulsby, C., Birkel, C., & Tetzlaff, D. (2014). Assessing urbanization impacts on catchment transit times. *Geophysical Research Letters*. 10.1002/2013GL058716

[CR73] Soulsby, C., Birkel, C., Geris, J., Tunaley, C., & Tetzlaff, D. (2015). Stream water age distributions controlled by storage dynamics and non-linear hydrologic connectivity: Modeling with high-resolution isotope data. *Water Resources Research,**51*, 7759–7776. 10.1002/2015WR017888 27478255 10.1002/2015WR017888 PMC4949550

[CR74] Stahl, K., Hisdal, H., Hannaford, J., Tallaksen, L. M., Van Lanen, H. A. J., Sauquet, E., Demuth, S., Fendekova, M., & J́odar, J. (2010). Streamflow trends in Europe: Evidence from a dataset of near-natural catchments. *Hydrology and Earth System Sciences,**14*(12), 2367–2382. 10.5194/hess-14-2367-2010

[CR75] Stevenson, J. L., Geris, J., Birkel, C., Tetzlaff, D., & Soulsby, C. (2022). Assessing land use influences on isotopic variability and stream water ages in urbanising rural catchments. *Isotopes in Environmental and Health Studies,**58*(3), 277–300. 10.1080/10256016.2022.2070615 35549960 10.1080/10256016.2022.2070615

[CR76] Strokal, M., Bai, Z., Franssen, W., Hofstra, N., Koelmans, A. A., Ludwig, F., Ma, L., van Puijenbroek, P., Spanier, J. E., Vermeulen, L. C., van Vliet, M. T. H., van Wijnen, J., & Kroeze, C. (2021). Urbanization: An increasing source of multiple pollutants to rivers in the 21st century. *Npj Urban Sustainability*. 10.1038/s42949-021-00026-w

[CR77] Szoszkiewicz, K., Achtenberg, K., Debbaut, R., Dekanova, V., Gebler, D., Jusik, S., Kaluza, T., Kartunnen, K., Lehti, N., Martin Munoz, S., Sojka, M., Pereira, A. J., Pinho, P., Schoelynck, J., Staes, J., Tetzlaff, D., Warter, M. M., & Vierrikko, K. (2024). Diversification of macrophytes within aquatic nature-based solutions (NBS) developing under urban environemntal conditions across European Cities. *Ecological Indicators, in Review*. 10.2139/ssrn.5068806

[CR78] Tabari, H., Taye, M. T., & Willems, P. (2015). Water availability change in central Belgium for the late 21st century. *Global and Planetary Change,**131*, 115–123. 10.1016/j.gloplacha.2015.05.012

[CR79] Tetzlaff, D., Seibert, J., McGuire, K. J., Laudon, H., Burns, D. A., Dunn, S. M., & Soulsby, C. (2009). How does landscape strucutre influence catchment transit time across different geomorphic provinces? *Hydrological Processes,**23*, 945–953. 10.1002/hyp

[CR80] Tetzlaff, D., Buttle, J., Carey, S. K., Mcguire, K., Laudon, H., & Soulsby, C. (2015). Tracer-based assessment of flow paths, storage and runoff generation in northern catchments: A review. *Hydrological Processes,**29*(16), 3475–3490. 10.1002/hyp.10412

[CR81] Tramblay, Y., Rutkowska, A., Sauquet, E., Sefton, C., Laaha, G., Osuch, M., Albuquerque, T., Alves, M. H., Banasik, K., Beaufort, A., Brocca, L., Camici, S., Csabai, Z., Dakhlaoui, H., DeGirolamo, A. M., Dörflinger, G., Gallart, F., Gauster, T., Hanich, L., & Datry, T. (2021). Trends in flow intermittence for European rivers. *Hydrological Sciences Journal,**66*(1), 37–49. 10.1080/02626667.2020.1849708

[CR82] van Rees, C. B., Jumani, S., Abera, L., Rack, L., McKay, S. K., & Wenger, S. J. (2023). The potential for nature-based solutions to combat the freshwater biodiversity crisis. *PLOS Water,**2*(6), Article e0000126. 10.1371/journal.pwat.0000126

[CR83] Vlaanderen, W. (2024). *Waterinfo Vlaanderen*. https://waterinfo.vlaanderen.be/Meetreeksen

[CR84] Von Freyberg, J., Allen, S. T., Seeger, S., Weiler, M., & Kirchner, J. W. (2018a). Sensitivity of young water fractions to hydro-climatic forcing and landscape properties across 22 Swiss catchments. *Hydrology and Earth System Sciences,**22*(7), 3841–3861. 10.5194/hess-22-3841-2018

[CR85] Von Freyberg, J., Studer, B., Rinderer, M., & Kirchner, J. W. (2018b). Studying catchment storm response using event- A nd pre-event-water volumes as fractions of precipitation rather than discharge. *Hydrology and Earth System Sciences,**22*(11), 5847–5865. 10.5194/hess-22-5847-2018

[CR86] von Freyberg, J., Rücker, A., Zappa, M., Schlumpf, A., Studer, B., & Kirchner, J. W. (2022). Four years of daily stable water isotope data in stream water and precipitation from three Swiss catchments. *Scientific Data,**9*(1), 1–10. 10.1038/s41597-022-01148-1 35145112 10.1038/s41597-022-01148-1 PMC8831500

[CR87] Vystavna, Y., Schmidt, S. I., Diadin, D., Rossi, P. M., Vergeles, Y., Erostate, M., Yermakovych, I., Yakovlev, V., Knöller, K., & Vadillo, I. (2019). Multi-tracing of recharge seasonality and contamination in groundwater: A tool for urban water resource management. *Water Research,**161*, 413–422. 10.1016/j.watres.2019.06.028 31226539 10.1016/j.watres.2019.06.028

[CR88] Walsh, C. J., Roy, A. H., Feminella, J. W., Cottingham, P. D., Groffman, P. M., & Morgan, R. P. (2005). The urban stream syndrome: Current knowledge and the search for a cure. *Journal of the North American Benthological Society,**24*(3), 706–723. 10.1899/04-028.1

[CR89] Walsh, C. J., Fletcher, T. D., & Burns, M. J. (2012). Urban stormwater runoff: A new class of environmental flow problem. *PLoS ONE*. 10.1371/journal.pone.0045814 10.1371/journal.pone.0045814 PMC3446928 23029257

[CR90] Wang, F., Tetzlaff, D., Goldhammer, T., Freymueller, J., & Soulsby, C. (2025). Hydrological connectivity drives intra- and inter-annual variation in water quality in an intermittent stream network in a mixed land use catchment under drought. *Journal of Hydrology*, *648*(August 2024), 132420. 10.1016/j.jhydrol.2024.132420

[CR91] Warter, M. M., Tetzlaff, D., Marx, C., & Soulsby, C. (2024b). Impact of drought hazards on flow regimes in anthropogenically impacted streams: An isotopic perspective on climate stress. *Natural Hazards and Earth System Science, 24,*1–18. 10.5194/nhess-24-3907-2024

[CR92] Warter, M. M., Tetzlaff, D., Ring, A. M., Christopher, J., Kissener, H. L., Funke, E., Sparmann, S., Mbedi, S., Soulsby, C., & Monaghan, M. T. (2024). Environmental DNA, hydrochemistry and stable water isotopes as integrative tracers of urban ecohydrology. *Water Research*, *250*. 10.1016/j.watres.2023.121065 10.1016/j.watres.2023.121065 38159541

[CR93] Warter, M. M., Tetzlaff, D., Soulsby, C., Goldhammer, T., Gebler, D., Vierrikko, K., & Monaghan, M. T. (2024d). Understanding ecohydrology and biodiversity in aquatic nature-based solutions in urban streams and ponds through an integrative multi-tracer approach. Hydrology and Earth System Sciences, in Review. 10.5194/egusphere-2024-3537

[CR94] Wild, T., Fuchs, G., & Davis, M. (2024). Sitting in our own soup ? Combined sewers, climate change and nature-based solutions for urban water management in Berlin. *Nature-Based Solutions,**5*, 100113. 10.1016/j.nbsj.2024.100113

[CR95] Williams, M. R., & Filoso, S. (2023). Changes in hydrology and pollutant loads from stream restoration in an urban headwater catchment. *Journal of Hydrology,**618*, Article 129164. 10.1016/j.jhydrol.2023.129164

[CR96] Wu, S., Tetzlaff, D., Goldhammer, T., & Soulsby, C. (2021). Hydroclimatic variability and riparian wetland restoration control the hydrology and nutrient fluxes in a lowland agricultural catchment. *Journal of Hydrology*, *603*(PB), 126904. 10.1016/j.jhydrol.2021.126904

[CR97] Yang, G., Bowling, L. C., Cherkauer, K. A., & Pijanowski, B. C. (2011). The impact of urban development on hydrologic regime from catchment to basin scales. *Landscape and Urban Planning,**103*(2), 237–247. 10.1016/j.landurbplan.2011.08.003

[CR98] Ying, Z., Tetzlaff, D., Freymueller, J., Comte, J. C., Goldhammer, T., Schmidt, A., & Soulsby, C. (2024). Developing a conceptual model of groundwater – Surface water interactions in a drought sensitive lowland catchment using multi-proxy data. *Journal of Hydrology*, *628*(December 2023), 130550. 10.1016/j.jhydrol.2023.130550

[CR99] Zanazzi, A., Wang, W., Peterson, H., & Emerman, S. H. (2020). Using stable isotopes to determine the water balance of Utah Lake (Utah, USA). *Hydrology,**7*(4), 1–25. 10.3390/hydrology7040088

