# Supplementary material for: Assessing the sensitivity of urban aquatic nature-based solutions to hydroclimate variability using stable water isotopes
Source: Environ Monit Assess. 2025 Dec 6;198(1):20. doi: 10.1007/s10661-025-14882-x (PMC12681493; doi:10.1007/s10661-025-14882-x)
Supplement: Supplementary file 1 — (DOCX 29.5 KB) [file 10661_2025_14882_MOESM1_ESM.docx]

**SUPPLEMENTARY MATERIAL: Assessing the sensitivity of urban aquatic nature-based solutions to hydroclimate variability using stable water isotopes**

Maria Magdalena Warter^1^, Chris Soulsby^2,3^, Kati Vierikko^4^, Silvia Martin Muñoz^5^, Daniel Gebler^6^, Mariusz Sojka^7^, Vladimíra Dekan Carreira^8^, Cristina Antunes^8^, Pedro Pinho^8^ and Dörthe Tetzlaff^129^

^1^ Leibniz Institute of Freshwater Ecology and Inland Fisheries, Department of Ecohydrology and Biogeochemistry, Berlin, Germany

^2^ Northern Rivers Institute, University of Aberdeen, St. Mary’s Building, Kings College, Old Aberdeen, Scotland

^3^ Chair of Water Resources Management and Modeling of Hydrosystems, Technical University Berlin, Berlin, Germany

^4^ Finnish Environment Institute, Built Environment Solutions Unit, Helsinki, Finland

^5^ Department of Biology, ECOSPHERE Research Group, University of Antwerp, Antwerp, Belgium

^6^ Department of Ecology and Environmental Protection, Poznań University of Life Sciences, Poland

^7^Department of Land Improvement, Environmental Development and Spatial Management, Poznań University of Life Sciences, Poland

^8^ cE3c - Center for Ecology, Evolution and Environmental Changes & CHANGE - Global Change and Sustainability Institute, Faculty of Sciences, University of Lisbon, C2, Campo Grande, Lisbon, Portugal

^9^ Department of Geography, Humboldt University of Berlin, Berlin, Germany

Table S1: Overview of sampling sites and sampling times in each city.

Table S2: Information on sampling sites including flow regime and storm discharge for streams and type, storm discharge and pond volume (in m^3^) for ponds.

Table S3: Results of DR and young water fractions for all aquaNBS sites.

**Table S1:** Overview of sampling times and number of samples in each city

| City | Sample date | Number of samples |
| --- | --- | --- |
| Poznań | Winter: February 2023  Spring: May 2023  Summer: July 2023  Autumn: November 2023 | n= 13  n= 13  n= 13  n= 13 |
| Berlin | Winter: February 2023  Spring: May 2023  Summer: August 2023  Autumn: November 2023 | n= 12  n= 12  n= 12  n= 12 |
| Antwerp | Winter: February 2023  Spring: April 2023  Summer: July 2023  Autumn: November 2023 | n=12  n=12  n=12  n=12 |
| Lisbon | Winter: February 2024  Spring: April 2023  Summer: July 2023  Autumn: November 2023 | n=12  n= 8 *some locations dry  n=12  n=12 |

**Table S2:** Information on sampling sites including flow regime and storm discharge for streams and type, storm discharge and surface area (in m^2^) for ponds.

| City | Site | Flow regime | Storm discharge | Effluent discharge | Volume  (m^3^) |
| --- | --- | --- | --- | --- | --- |
|  | **Stream-based NBS** | | | | |
| Poznań | PL01 | Perennial | yes | yes |  |
|  | PL02 | Perennial | yes | no |  |
|  | PL03 | Intermittent | yes | no |  |
|  | PL04 | Intermittent | yes | no |  |
|  | PL05 | Perennial | yes | no |  |
|  | PL13 | Perennial | yes | yes |  |
| Berlin | DE02 | Intermittent | yes | yes |  |
|  | DE03 | Perennial | yes | yes |  |
|  | DE04 | Perennial | yes | yes |  |
|  | DE07 | Intermittent | yes | no |  |
|  | DE09 | Intermittent | yes | no |  |
|  | DE10 | Perennial | yes | yes |  |
|  | DE11 | Perennial | yes | yes |  |
|  | DE12 | Perennial | yes | no |  |
| Lisbon | PT01 | Perennial | no | no |  |
|  | PT02 | Perennial | no | no |  |
|  | PT03 | Perennial | no | no |  |
|  | PT04 | Perennial | yes | no |  |
|  | PT05 | Intermittent | yes | no |  |
|  | PT06 | Intermittent | yes | no |  |
|  | PT07 | Intermittent | yes | no |  |
|  | **Pond-based NBS** | | | | |
| Poznań | PL06 | Intermittent | yes | yes | 17500 |
|  | PL07 | Perennial | yes | yes | 8000 |
|  | PT08 | Perennial | yes | no | 3300 |
|  | PT10 | Perennial | yes | no | 25500 |
|  | PT11 | Intermittent | yes | no | 32000 |
|  | PT12 | Perennial | yes | no | 1350 |
| Berlin | DE01 | Perennial | yes | no | 6000 |
|  | DE05 | Perennial | yes | no | 55130 |
|  | DE06 | Perennial | no | no | 107420 |
|  | DE08 | Perennial | no | no | 15600 |
| Antwerp | BE01 | Intermittent | yes | no | 1651 |
|  | BE02 | Intermittent | yes | no | 54 |
|  | BE03 | Perennial | yes | no | 1668 |
|  | BE04 | Perennial | yes | no | 308 |
|  | BE05 | Perennial | yes | no | 810 |
|  | BE06 | Perennial | yes | no | 2068 |
|  | BE07 | Perennial | yes | no | 810 |
|  | BE08 | Intermittent | yes | no | 732 |
|  | BE09 | Intermittent | yes | no | 84 |
|  | BE10 | Perennial | yes | no | 2140 |
|  | BE11 | Perennial | yes | no | 2140 |
|  | BE12 | Intermittent | yes | no | 224 |
| Lisbon | PT08 | Perennial | yes | no | 20000 |
|  | PT09 | Perennial | yes | no | 2200 |
|  | PT10 | Perennial | yes | no | 2760 |
|  | PT11 | Perennial | no | no | 2890 |
|  | PT12 | Perennial | no | no | 84 |

**Table S3:** Results of DR and young water fractions for all aquaNBS sites.

|  | DR | | F_yw_ | | Type of aquaNBS |  |
| --- | --- | --- | --- | --- | --- | --- |
|  | δ18O | δ2H | δ18O | δ2H |  |  |
| Poznań | | | | | | |
| PL01 | *0.32* | *0.3* | *0.38* | *0.34* | *Stream* |  |
| PL02 | *0.13* | *0.1* | *0.19* | *0.13* | *Stream* |  |
| PL03 | *0.17* | *0.15* | *0.22* | *0.18* | *Stream* |  |
| PL04 | *0.63* | *0.63* | *0.99* | *0.95* | *Stream* |  |
| PL05 | *0.15* | *0.12* | *0.22* | *0.17* | *Stream* |  |
| PL06 | *0.3* | *0.24* | *0.38* | *0.3* | *Pond* |  |
| PL07 | *0.46* | *0.45* | *0.57* | *0.5* | *Pond* |  |
| PL08 | *0.66* | *0.65* | *0.77* | *0.66* | *Pond* |  |
| PL09 | *0.7* | *0.65* | *0.88* | *0.75* | *Pond* |  |
| PL10 | *0.27* | *0.23* | *0.24* | *0.21* | *Pond* |  |
| PL11 | *0.39* | *0.25* | *0.34* | *0.24* | *Pond* |  |
| PL12 | *0.89* | *0.65* | *0.99* | *0.77* | *Pond* |  |
| Berlin | | | | | | |
| DE01 | *0.94* | *0.36* | *0.55* | *0.35* | *Pond* |  |
| DE02 | *0.43* | *0.24* | *0.49* | *0.3* | *Stream* |  |
| DE03 | *0.08* | *0.06* | *0.09* | *0.07* | *Stream* |  |
| DE04 | *0.26* | *0.19* | *0.28* | *0.24* | *Stream* |  |
| DE05 | *0.37* | *0.19* | *0.35* | *0.24* | *Pond* |  |
| DE06 | *0.29* | *0.13* | *0.29* | *0.18* | *Pond* |  |
| DE07 | *0.41* | *0.43* | *0.43* | *0.42* | *Stream* |  |
| DE08 | *0.38* | *0.31* | *0.6* | *0.5* | *Pond* |  |
| DE09 | *0.48* | *0.5* | *0.62* | *0.66* | *Stream* |  |
| DE10 | *0.17* | *0.03* | *0.27* | *0.06* | *Stream* |  |
| DE11 | *0.2* | *0.18* | *0.15* | *0.2* | *Stream* |  |
| DE12 | *0.22* | *0.23* | *0.19* | *0.27* | *Stream* |  |
| Antwerp | | | | | | |
| BE01 |  | | *>1.0* |  | *Pond* |  |
| BE02 |  | | *0.98* |  | *Pond* |  |
| BE03 |  | | *>1.0* |  | *Pond* |  |
| BE04 |  | | *>1.0* |  | *Pond* |  |
| BE05 |  | | *>1.0* |  | *Pond* |  |
| BE06 |  | | *>1.0* |  | *Pond* |  |
| BE07 |  | | *>1.0* |  | *Pond* |  |
| BE08 |  | | *>1.0* |  | *Pond* |  |
| BE09 |  | | *>1.0* |  | *Pond* |  |
| BE10 |  | | *>1.0* |  | *Pond* |  |
| BE11 |  | | *>1.0* |  | *Pond* |  |
| BE12 |  | | *>1.0* |  | *Pond* |  |
| Lisbon | | | | | | |
| PT01 |  |  |  | |  |  |
| PT02 | *0.33* | *0.17* | *0.38* | *0.72* | *Stream* |  |
| PT03 | *0.14* | *0.03* | *0.18* | *0.04* | *Stream* |  |
| PT04 | *0.15* | *0.17* | *0.15* | *0.22* | *Stream* |  |
| PT05 | *0.26* | *0.12* | *0.24* | *0.14* | *Stream* |  |
| PT06 | *0.08* | *0.14* | *0.08* | *0.18* | *Stream* |  |
| PT07 | *0.11* | *0.14* | *0.14* | *0.23* | *Stream* |  |
| PT08 | *0.71* | *0.37* | *0.68* | *0.43* | *Stream* |  |
| PT09 | *1.3* | *0.56* | *1.1* | *0.63* | *Pond* |  |
| PT10 | *0.21* | *0.11* | *0.25* | *0.13* | *Pond* |  |
| PT11 | *0.43* | *0.39* | *0.37* | *0.41* | *Pond* |  |
| PT12 | *4.9* | *1.69* | *nan* | *nan* | *Pond* |  |
